# Supplementary material for: CoSMIC: A hybrid approach for large-scale, high-resolution microbial profiling of novel niches
Source: PLoS One. 2026 Jan 27;21(1):e0340349. doi: 10.1371/journal.pone.0340349 (PMC12844514; doi:10.1371/journal.pone.0340349)
Supplement: CoSMIC_Supp.pdf — (PDF) [file pone.0340349.s001.pdf]

Supplementary data

Code is available at:
https://github.com/NoamShental/CoSMIC
Raw reads are available at:
https://www.dropbox.com/sh/kyaecrfnpkdxd96/AACANythrtzEBQApCalFzwUza?dl=0

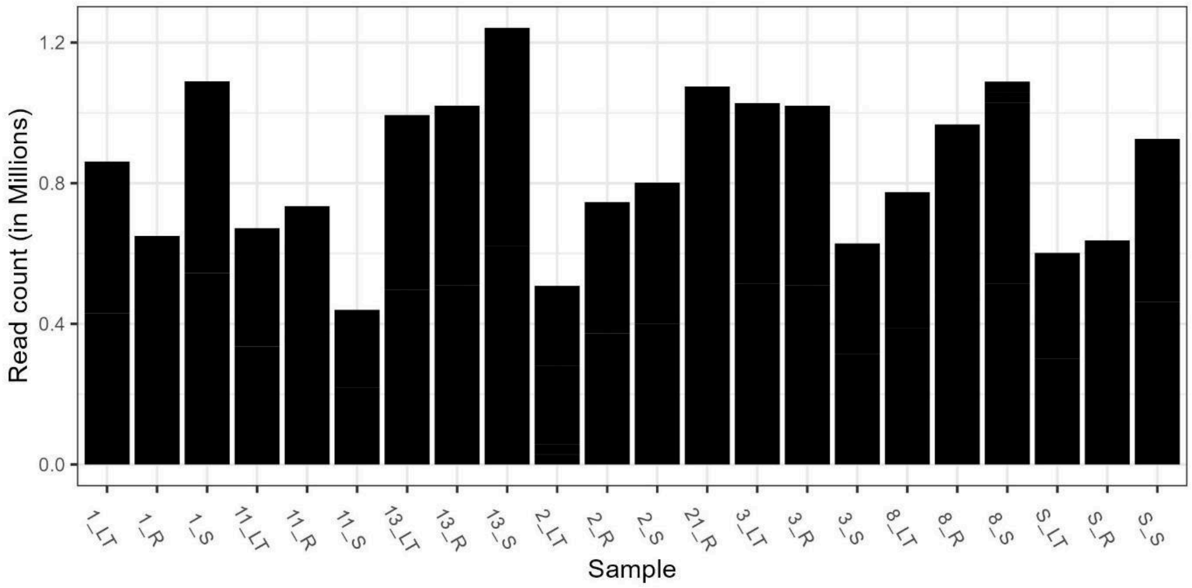

Figure S1. Number of Illumina reads for Qiagen QIAseq sequenced samples. The number of raw Illumina reads for each sample prepared using the Qiagen QIA seq kit, which amplifies six variable regions along the 16S rRNA gene. Sample descriptions appear in Table S1 using the same abbreviations as the horizontal axis.

Figures S2-6

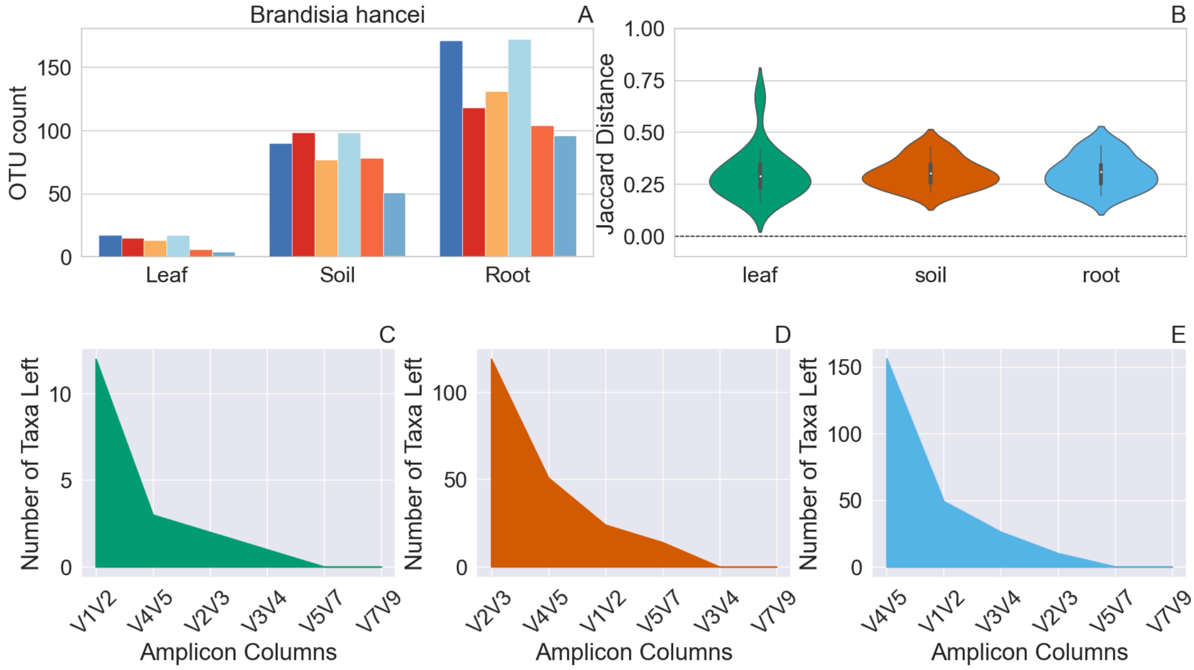

Figure S2. Microbial profiling of *Brandisia hancei* depends on the applied amplicon. Leaf, soil, and root samples were profiled using the Qiagen kit that provides six amplicons along the 16S rRNA gene, and their reads were subsequently analyzed using the CLC Genomics platform. **A.** The number of OTUs detected for soil, leaf, and root samples of each plant for each of the six amplicons. **B.** Distribution of Jaccard distances among pairs of amplicons. In each pair, e.g., V1V2 vs. V3V4, a Jaccard distance is calculated between the lists of taxons detected by CLC Genomics in each region. **C-E.** Decay graph of the number of unique taxons identified by the amplicons analyzed. Amplicons are ordered by the number of unique taxons a region contributes to the former set of regions. C-Leaf, D-Soil, and E-Root.

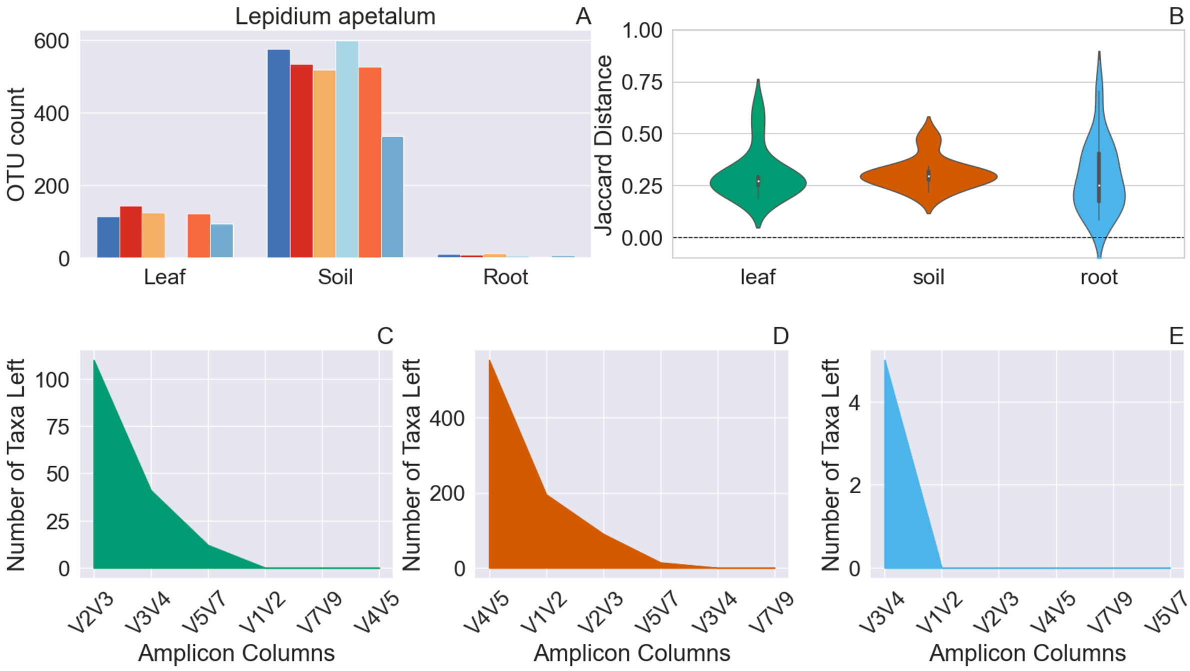

Figure S3. Analogous figure to Fig. S1, for *Lepidium apetalum*

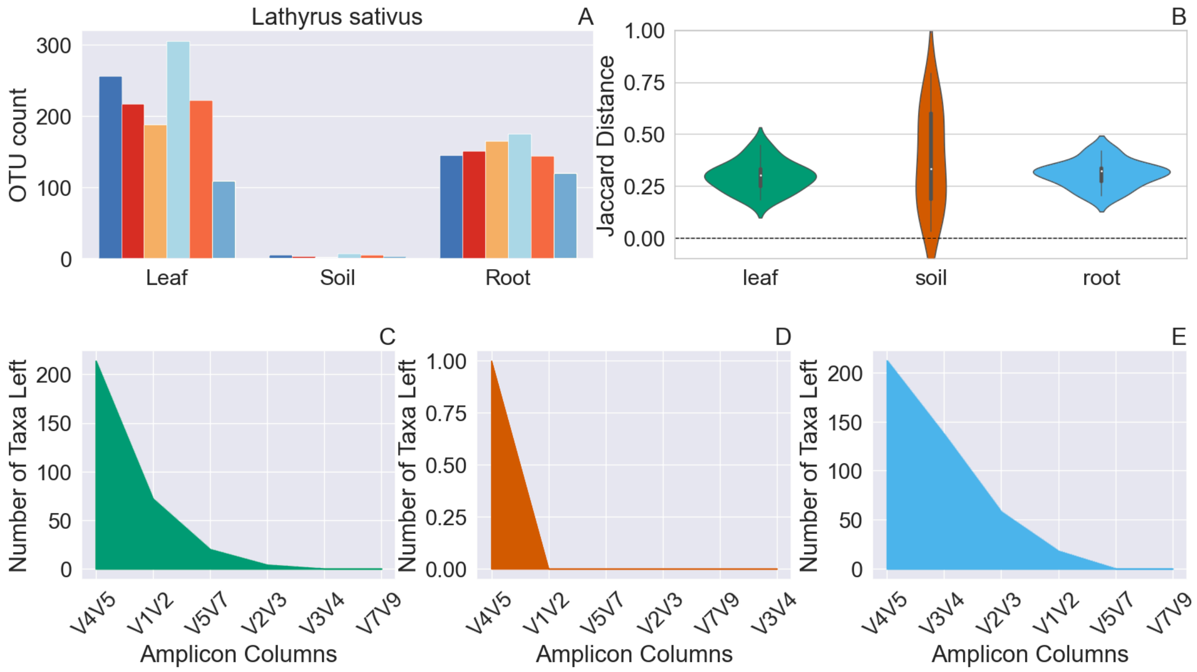

Figure S4. Analogous figure to Fig. S1, for *Lathyrus sativus*

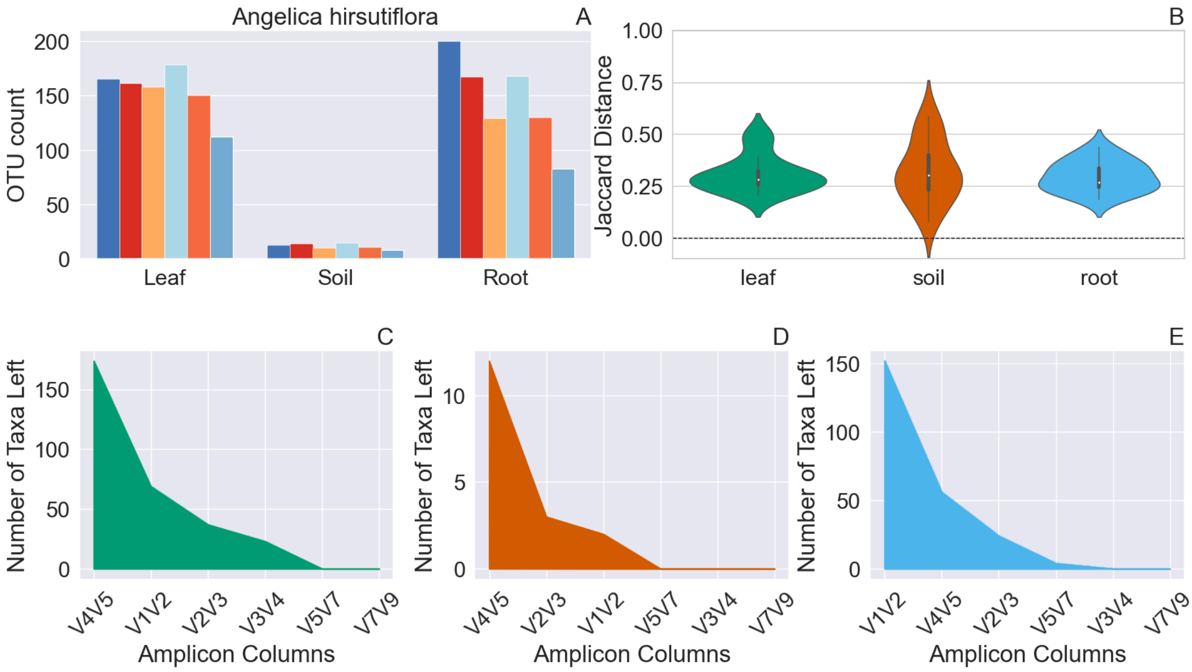

Figure S5. Analogous figure to Fig. S1, for *Angelica hirsutiflora*

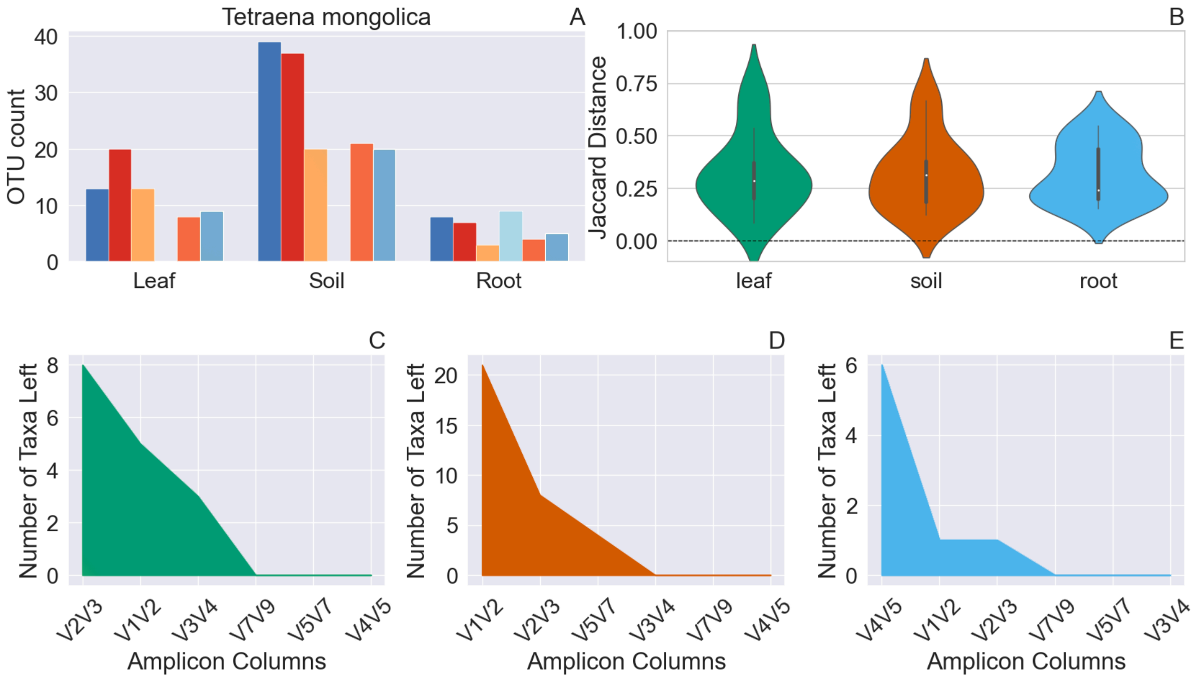

Figure S6. Analogous figure to Fig. S1, for *Tetraena mongolica*

Figures S7-9

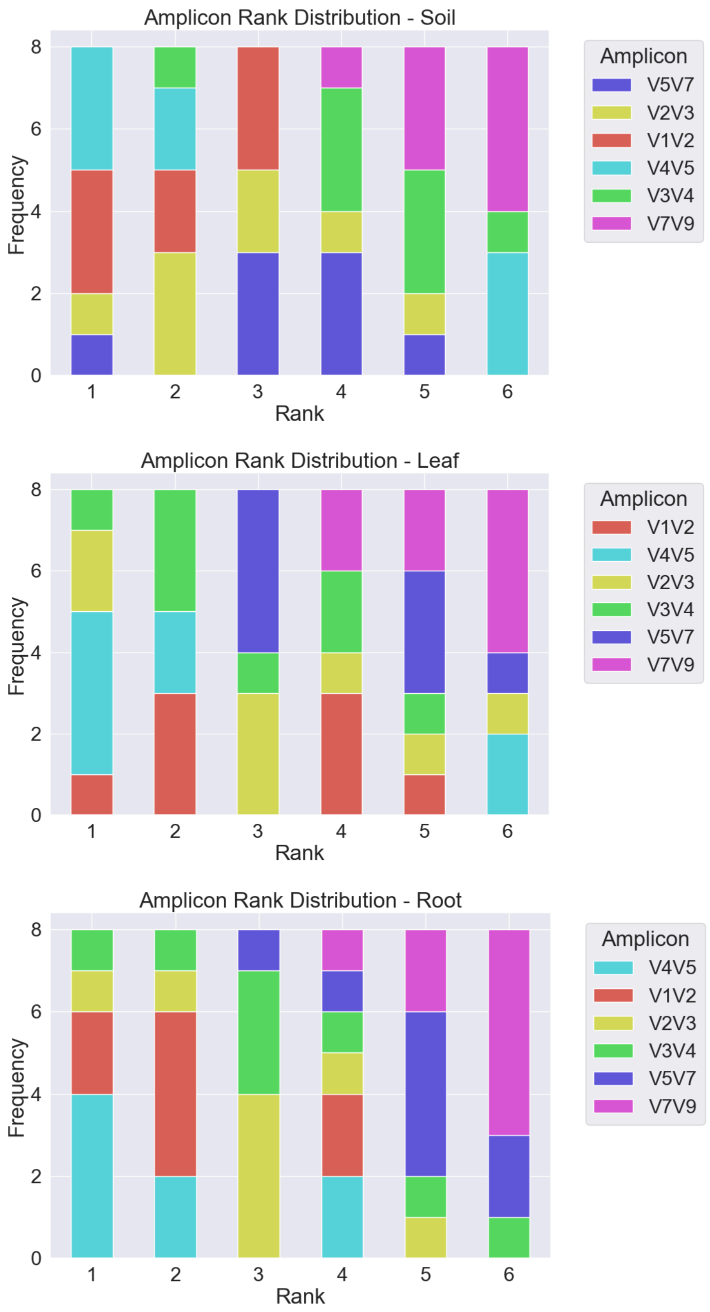

Figures S7-9. Ranking of Amplicons used in Qiagen analysis. Analysis of leaf, soil, and root samples involved six distinct amplicons. In this figure, each amplicon is ranked according to the amount of information it contributed to the analysis. There is no evident ranking, thus indicating that the information contribution for each measurement originates from varying amplicons.

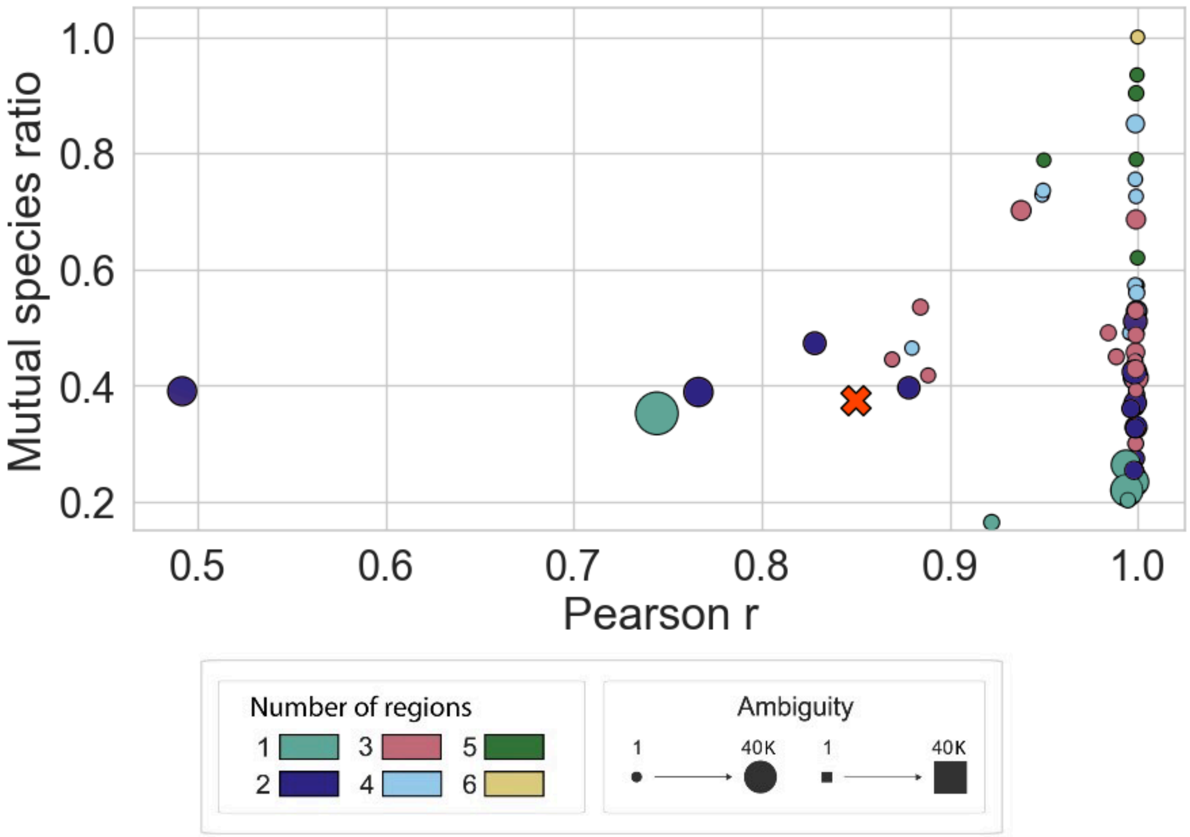

Figure S10. A comparison of amplicon sets for leaf samples. Each dot corresponds to a specific region combination averaged across samples, where the marker size scales with the ambiguity and axes correspond to Pearson correlation (horizontal) and mutual species (vertical). A marker's color indicates the number of amplicons used for SMURF analysis. Hence, a single marker corresponds to six regions (yellow), and six green dots correspond to the different groups of five regions. The marker "x" corresponds to a specific set of three regions comprising V1V2, V2V3, and V4V5.

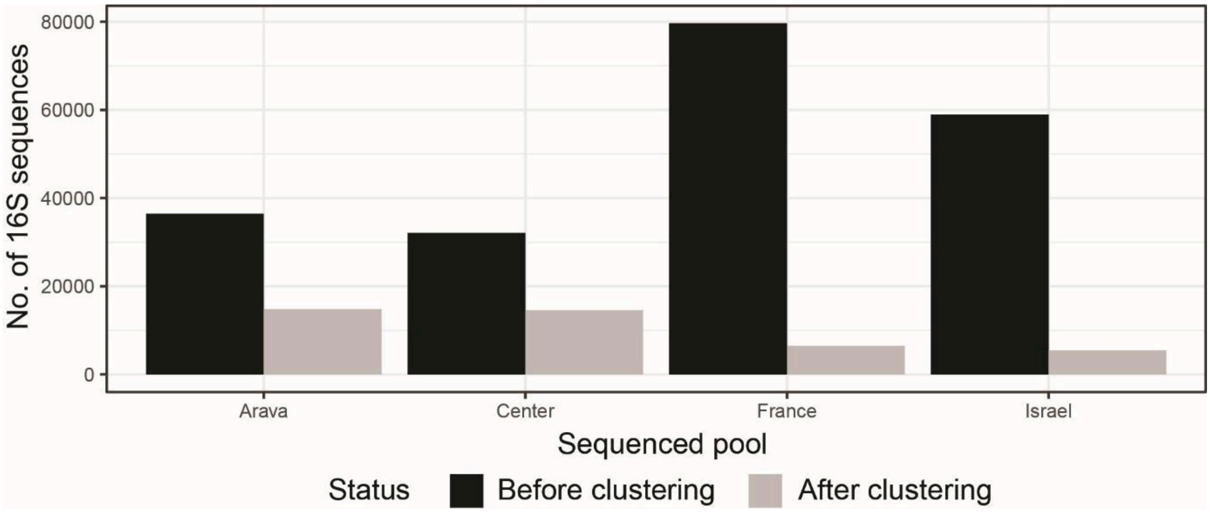

Figure S11. Number of 16S rRNA gene sequences derived from PacBio long-read sequencing of pooled samples. To reduce the redundancy of amplicons derived from the PacBio long-read sequencing, we clustered each sample using CD-HIT with an identity threshold of 99%. In black are the number of sequences before clustering; in gray are the number of seeds obtained after clustering. Sample descriptions appear in Table S1 using the same abbreviations as the horizontal axis.

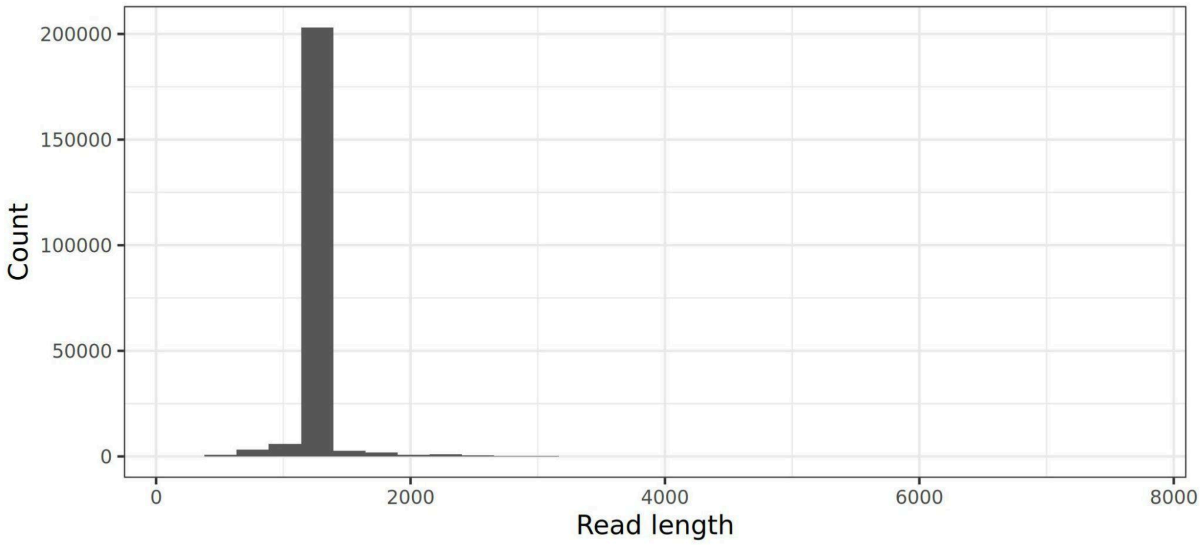

Figure S12. Read length distribution for PacBIo long reads sequences. Read length distribution of LNA-based 16S rRNA amplicons, obtained from the LNA amplification of all four pools of samples (*Spongia officinalis* from France and Israel; and plants from Israel's lowland and Arava regions).

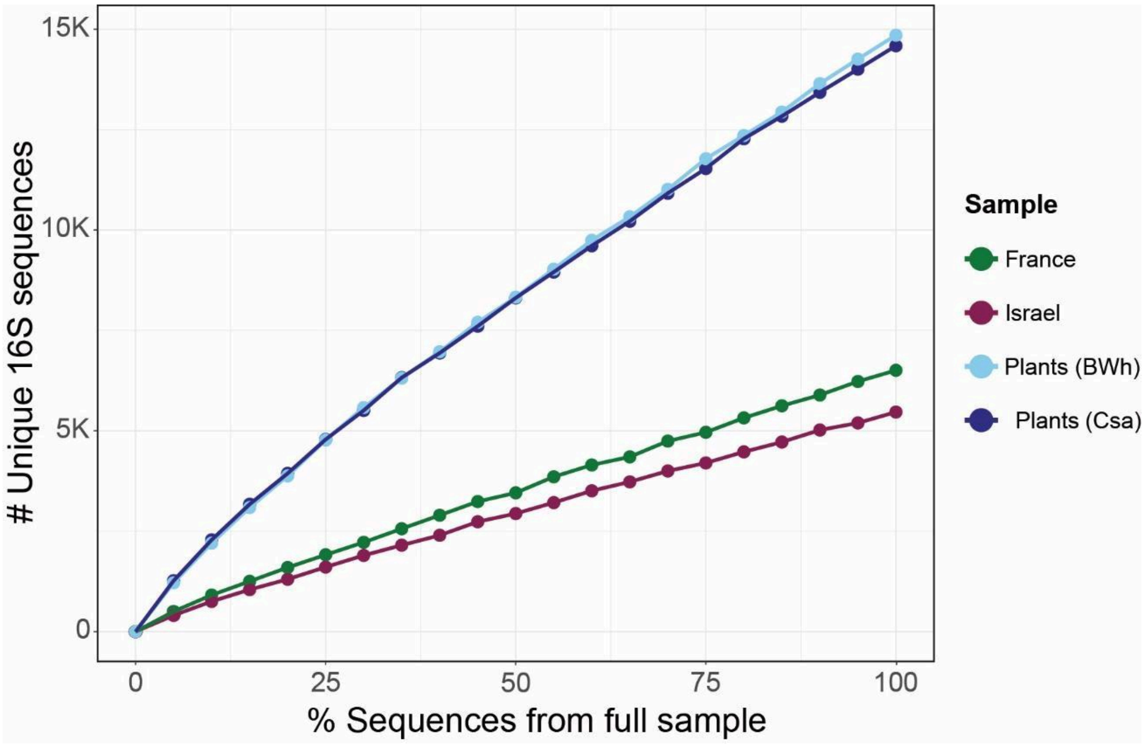

Figure S13. Rarefaction curves presenting the number of novel full-length 16S rRNA sequences identified (y-axis) based on the percentage of reads sampled (x-axis) for each of the sequenced pools

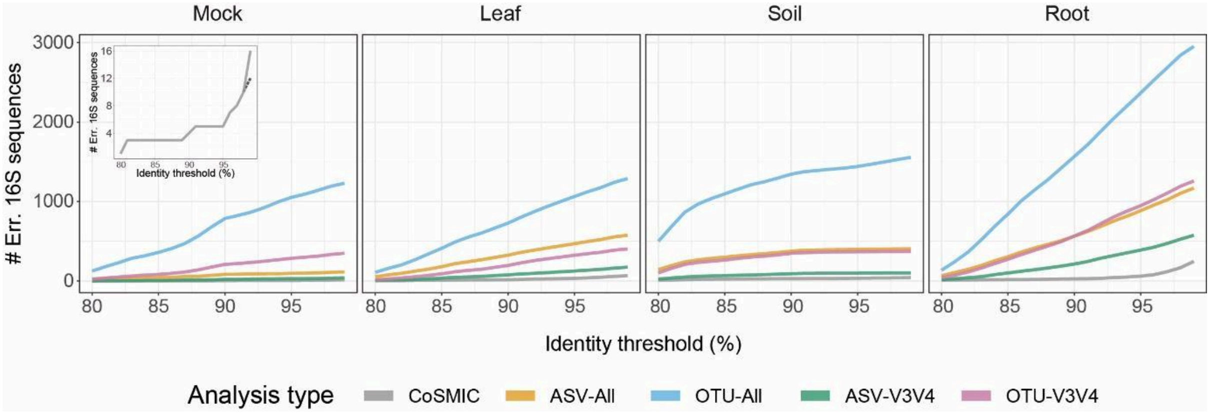

Figure S14. The absolute number of erroneous 16S rRNA gene sequences. A supplementary figure to Fig. 5C shows the number of erroneous 16S rRNA gene sequences detected by each method for each sample type as a function of the identity threshold. Erroneous sequences are those that were identified by each method yet were not detected by shotgun metagenomics for each identity threshold. The inset for the Mock mixture (left panel) zooms into CoSMIC's results. The dotted line corresponds to CoSMIC's results for up to a 99% identity threshold when improving the ground truth via MarkerMag.

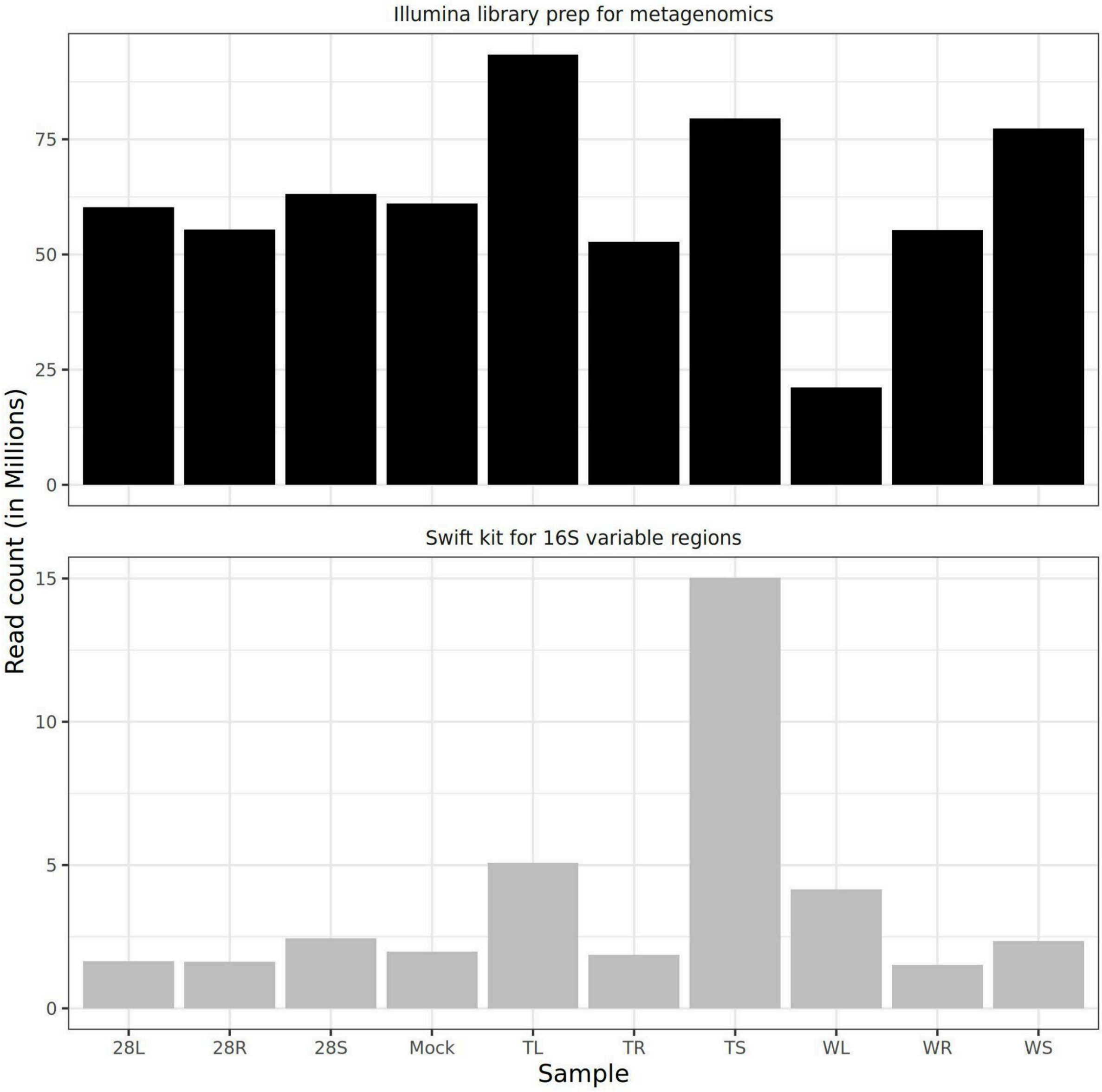

Figure S15. Number of Illumina reads obtained for each sample. For each sample (horizontal), the number of raw reads obtained by Illumina short-read sequencing is presented on the vertical axis (in millions). The upper panel corresponds to samples prepared for metagenomics analysis, while the lower panel corresponds to samples prepared using the Swift Biosciences kit, which amplifies multiple regions of the 16S rRNA gene. Sample descriptions appear in Table S1 using the same abbreviations as the horizontal axis.

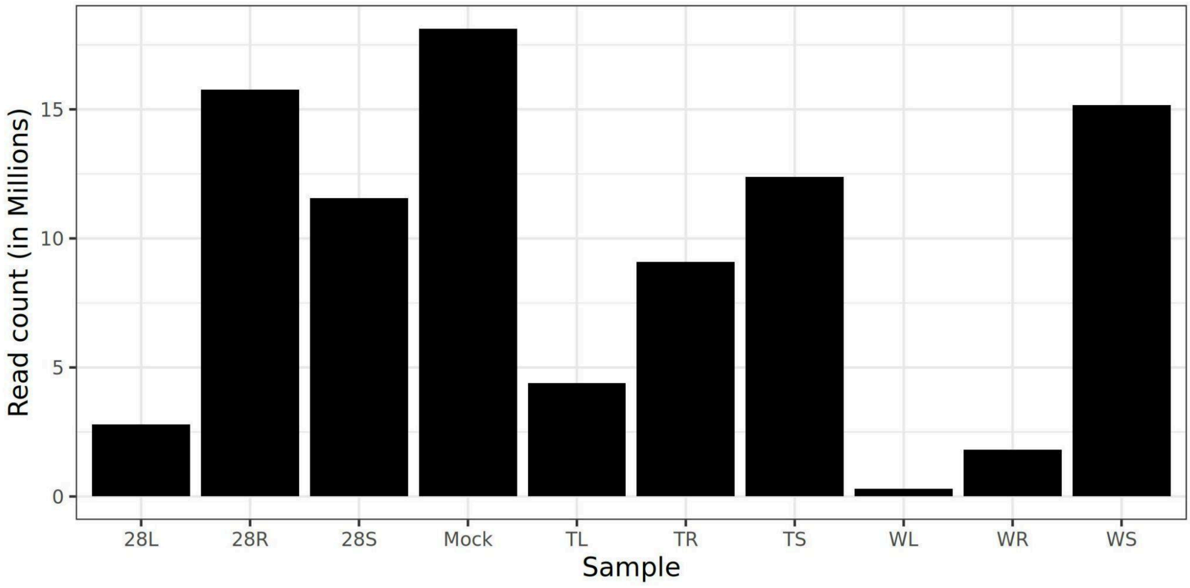

Figure S16. Number of reads for samples sequenced using Loop Genomics synthetic long reads. Sample descriptions appear in Table S1 using the same abbreviations as the horizontal axis.

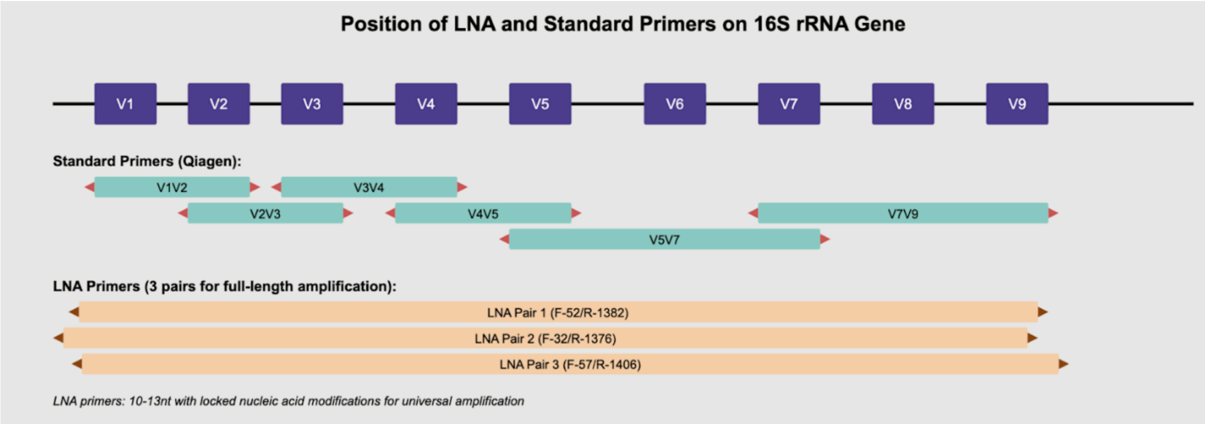

Figure S17. Positions of LNA and standard primers along the bacterial 16S rRNA gene. Schematic representation of variable regions (upper panel) and primer locations for six pairs included in the Qiagen QIAseq panel (middle panel). The lower panel shows the location of our LNA primers, positioned at conserved regions near the gene termini (forward primers at positions 32-57; reverse primers at positions 1376-1406 relative to *E. coli* 16S rRNA).

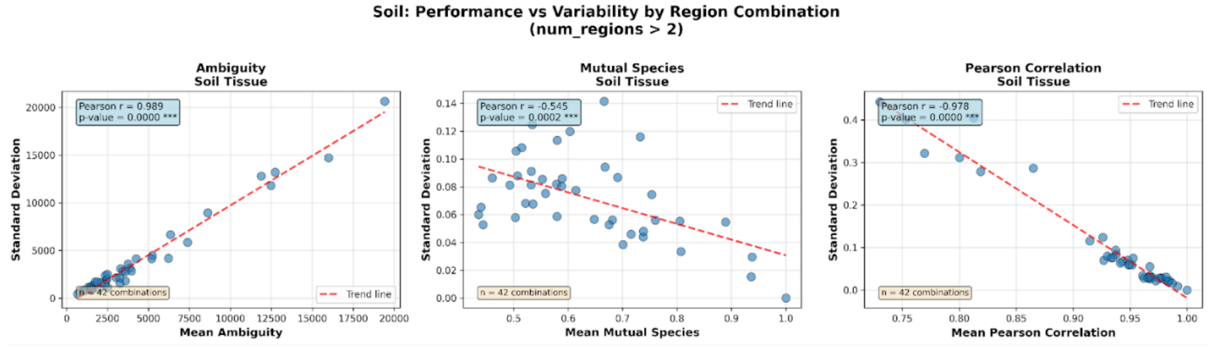

Figure S18: Performance measures of each primer pair combination - the correlation between the mean value across soil samples and their variance. We analyzed all 42 combinations of 2, 3, 4, and 5 primer pairs and calculated Ambiguity (left), Mutual Species (middle), and Pearson correlation (right). For each primer combination, we compared the mean of the relevant measure across 8 soil samples (horizontal axis) to its variance. Red dashed lines indicate linear regression trends. Statistical annotations show Pearson correlation coefficients (r) with corresponding p-values, where significance is indicated as: *** p < 0.001, ** p < 0.01, * p < 0.05, ns = not significant.

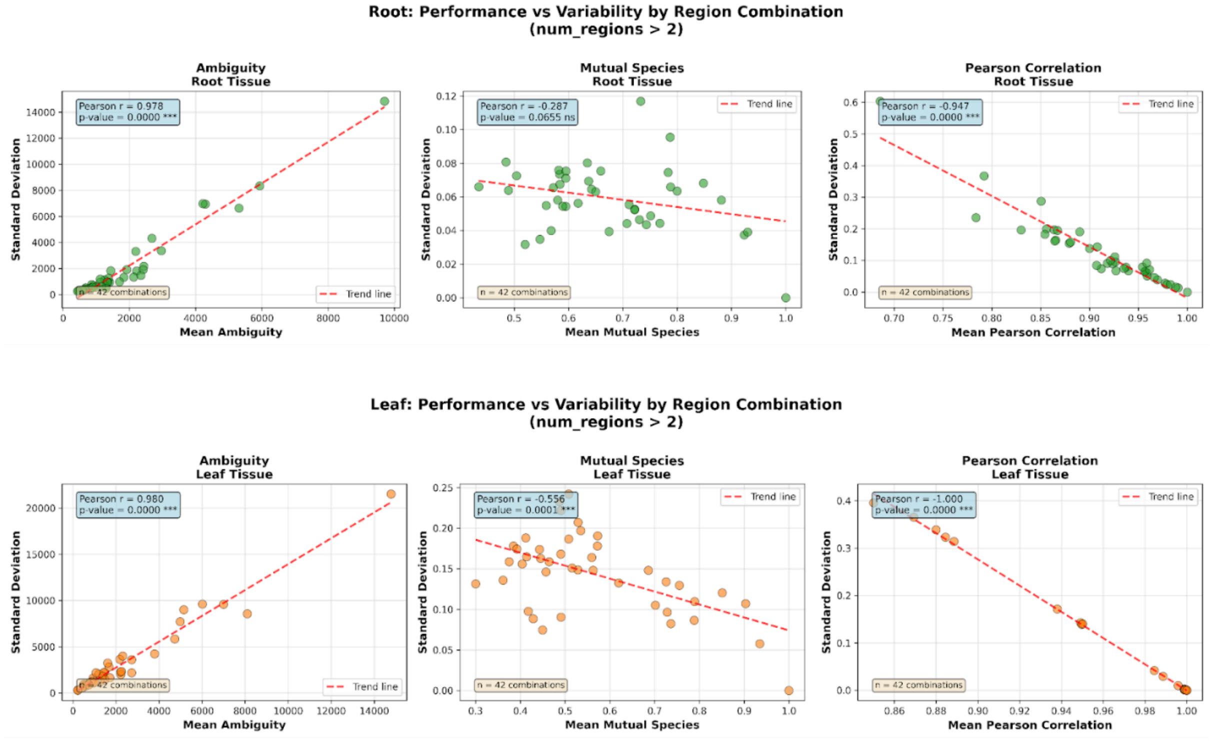

Figure S19: The same as Figure S18, for root (upper panel) and leaf (lower panels)

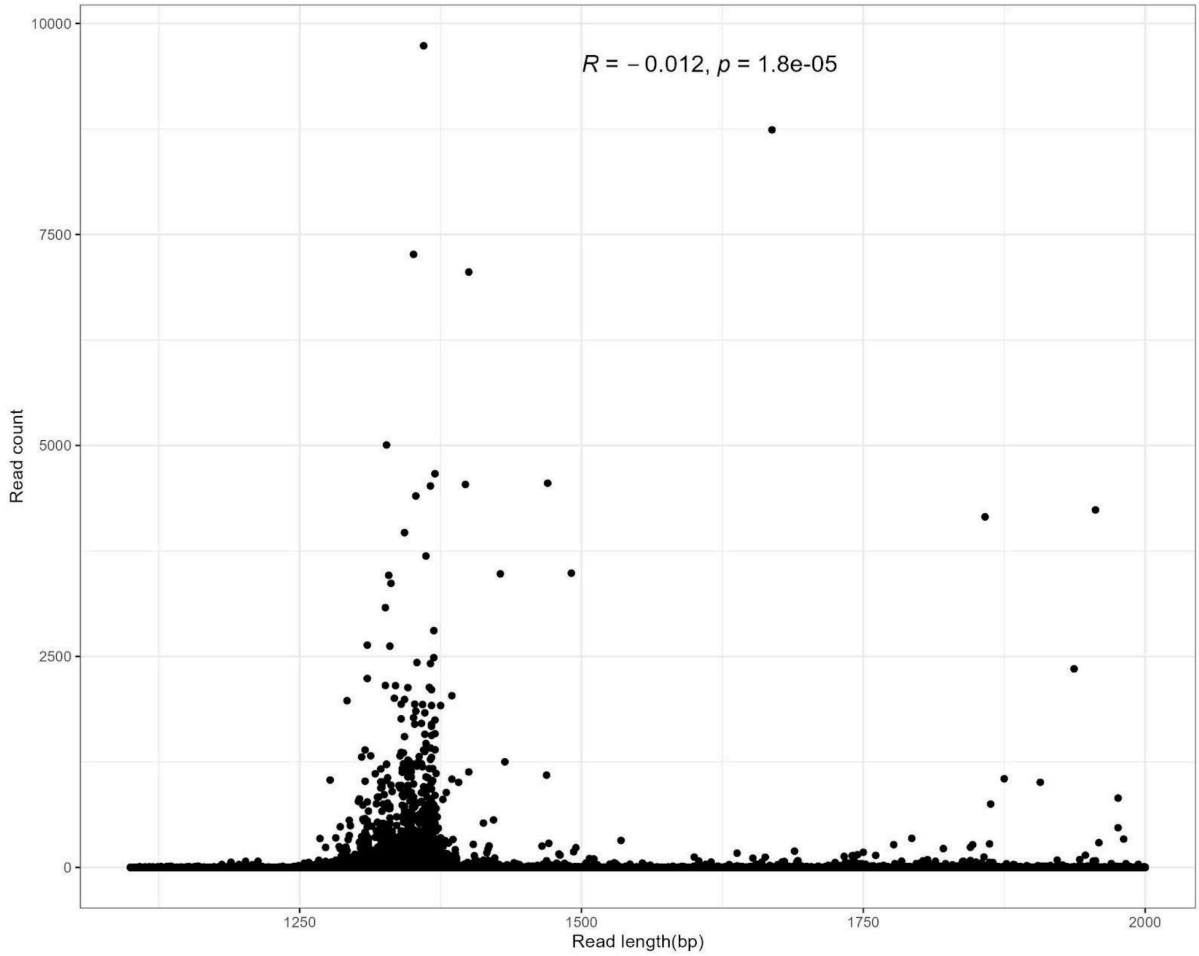

Figure S20. Read count as a function of read length for PacBio sequences retained in the database. Each point corresponds to a 16S rRNA sequence (or a cluster of sequences) retained after PacBio sequencing and subsequent postprocessing and filtering. The figure displays each sequence length vs. its observed count. There is no evidence of an association between sequence length and abundance (Pearson R = −0.012, p = 1.8e-5).

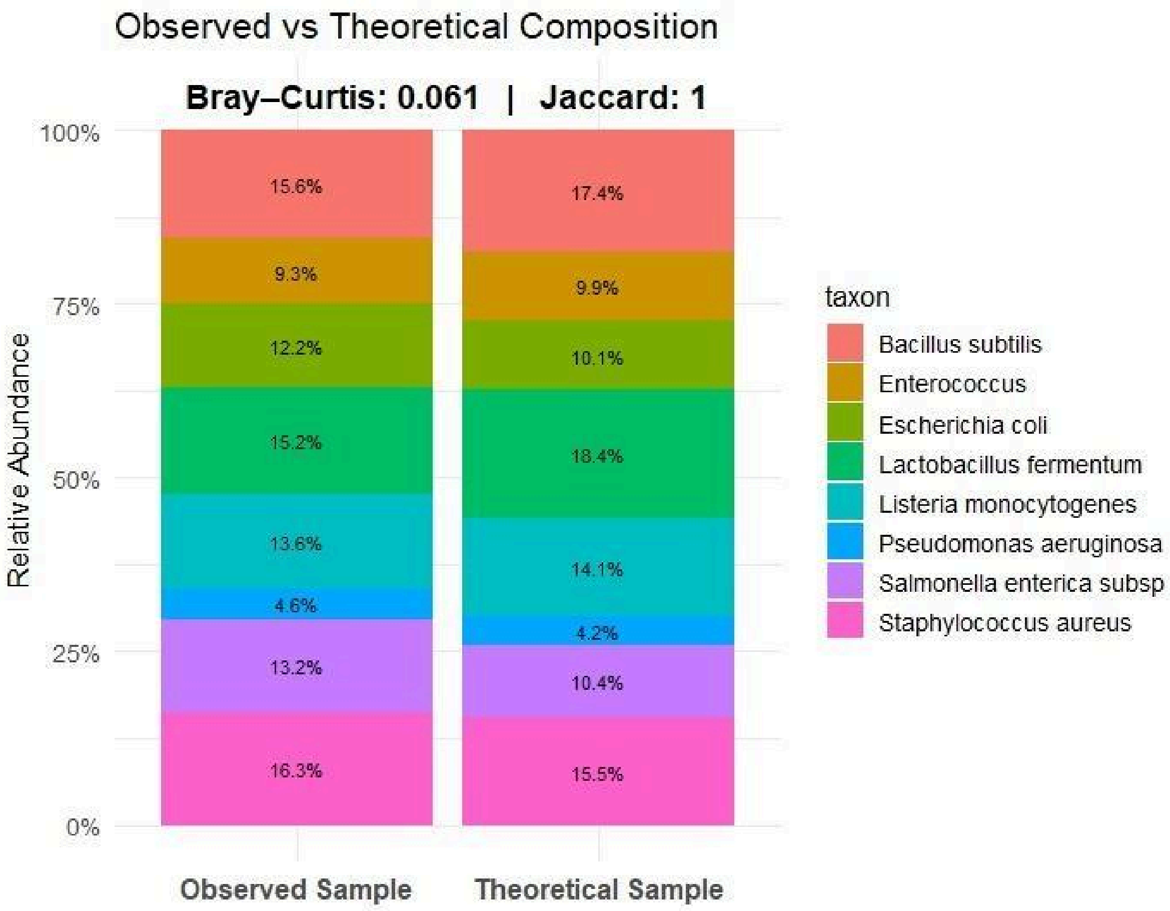

Figure S21. CoSMIC-based profiling of the ZymoBIOMICS microbial community DNA standard. The stacked bar charts compare the microbial composition of the ZymoBIOMICS standard (Zymo Research, Cat. No. D6305) as profiled by the CoSMIC pipeline (Observed Sample) versus the manufacturer's specified composition (Theoretical Sample). Each colored segment indicates the relative abundance of a specific taxon. A Jaccard index of 1.0 indicates that CoSMIC accurately identified all eight expected bacterial species with no false positives. The low Bray-Curtis dissimilarity score of 0.061 reflects a high similarity between the observed and theoretical relative abundances. Together, these findings support the accuracy of the CoSMIC framework against a well-defined, standardized benchmark.

Table S1. List of samples used in the study. The following abbreviations are used: *Organism* - the organism from which the sample was taken; *Tissue* - the tissue sampled in the organism; *Sample ID* - the sample name as recorded in our data; *Sample preparation kit* - the commercial kit used to prepare the samples for microbiome analysis; *Relevant results section* - the relevant Results section; *Description* - whether a single sample or a pool of samples. An asterisk refers to pools of multiple samples.

Organism	Tissue	Sample ID	Sample preparation kit	Relevant results section	Description
Lepidium apetalum	Soil	1_S	Qiagen QIAseq	Combining results from several variable regions partially overcomes problems in profiling	Single sample
	Leaf	1_LT	Qiagen QIAseq		Single sample
	Root	1_R	Qiagen QIAseq		Single sample
Lepidium apetalum	Soil	2_S	Qiagen QIAseq		Single sample
	Leaf	2_LT_5	Qiagen QIAseq		Single sample
	Leaf	2_LT_8	Qiagen QIAseq		Single sample
	Root	2_R	Qiagen QIAseq		Single sample
Aster poliothamnus	Soil	3_S	Qiagen QIAseq		Single sample
	Leaf	3_LT	Qiagen QIAseq		Single sample
	Root	3_R	Qiagen QIAseq		Single sample
Brandisia hancei	Soil	8_S_1	Qiagen QIAseq		Single sample
	Soil	8_S_4	Qiagen QIAseq		Single sample
	Leaf	8_LT	Qiagen QIAseq		Single sample
	Root	8_R	Qiagen QIAseq		Single sample
Tetraena mongolica	Soil	11_S	Qiagen QIAseq		Single sample
	Leaf	11_LT	Qiagen QIAseq		Single sample
	Root	11_R	Qiagen QIAseq		Single sample
Angelica hirsutiflora	Soil	13_S	Qiagen QIAseq		Single sample
	Leaf	13_LT	Qiagen QIAseq		Single sample
	Root	13_R	Qiagen QIAseq		Single sample

	Root	21_R	Qiagen QIAseq		Single sample
Lathyrus sativus	Soil	S_S	Qiagen QIAseq		Single sample
	Leaf	S_LT	Qiagen QIAseq		Single sample
	Root	S_R	Qiagen QIAseq		Single sample
Hymelaea hirsuta	Soil	28S	Swift 16S+ITS PANEL	Experimental evaluation of CoSMIC	Single sample
	Leaf	28L	Swift 16S+ITS PANEL		Single sample
	Root	28R	Swift 16S+ITS PANEL		Single sample
Solanum lycopersicum	Soil	TS	Swift 16S+ITS PANEL		Single sample
	Leaf	TL	Swift 16S+ITS PANEL		Single sample
	Root	TR	Swift 16S+ITS PANEL		Single sample
Triticum aestivum	Soil	WS	Swift 16S+ITS PANEL		Single sample
	Leaf	WL	Swift 16S+ITS PANEL		Single sample
	Root	WR	Swift 16S+ITS PANEL		Single sample
Mock community	Mock	Mock	Swift 16S+ITS PANEL		Mock community built from 12 known species (listed in the methods section)
Spongia officinalis - France*		France	PacBio SMRTbell Express template Prep Kit 2.0	Enriching the database using long-read sequencing	Pooled samples (seven) from the Mediterranean sea in Marseille, France*
Spongia officinalis - Israel*		Israel	PacBio SMRTbell Express template Prep Kit 2.0		Pooled samples (three) from the Mediterranean sea near Haifa, Israel*
Arava*		Arava	PacBio SMRTbell Express template Prep Kit 2.0		Pooled plant samples (33) from the desert region*
Lowlands pool*		Center	PacBio SMRTbell Express template Prep Kit 2.0		pooled plant samples (21) from the lowlands region*
Loop pool*		Loop_LNA	Loop genomics LoopSeq PCR Amplicon Kit		Pooled plant samples (11) used in Qiagen

					and Swift Analysis*

Analysis of No-Template Controls (NTCs)

To evaluate the potential effects of reagent and environmental contamination, eleven no-template controls (NTCs) were included and processed alongside the experimental samples across various sequencing libraries. Raw sequencing data from these NTC libraries produced a median of 22,875 reads per sample (Interquartile Range [IQR]: 2,493–43,637; Full Range: 1–169,257). Although raw reads were present, they were effectively removed during our bioinformatics pipeline. A detailed analysis of a representative NTC sample (11,425 reads) showed that the reads consisted of Illumina adapter sequences and very short DNA fragments (~35 bp). Due to their short length and low quality, these sequences were filtered out. As a result, no valid 16S rRNA sequences from any NTC passed to SMURF, leading to empty community profiles for all negative controls. This demonstrates that our workflow is resilient to low-level contamination and that such artifacts do not affect the microbial profiles reported in this study.
