# Supplementary material for: CoSMIC: A hybrid approach for large-scale, high-resolution microbial profiling of novel niches
Source: PLoS One. 2026 Jan 27;21(1):e0340349. doi: 10.1371/journal.pone.0340349 (PMC12844514; doi:10.1371/journal.pone.0340349)
Supplement: Attachment — Submitted filename: Knafo & Rezenman et al CoSMIC rebuttal letter 2025.pdf [file pone.0340349.s002.pdf]

Remark

Original comments from the Editor and Reviewers appear in *slanted* font; Our response appears in regular font; whenever we cite from the manuscript, the original text appears in **black,** while the revised text is in **blue**.

Editorial Remarks for Author:

"If you choose to revise this work according to the reviewers comments, please ensure that there is clarity in the description and purpose of CoSMIC. Inclusion of code or other information that would allow replication by others would also be helpful."

A: We thank the Editor for these remarks. In summary, the revised manuscript addresses the following points:

- The manuscript now clearly describes CoSMIC and its purpose. The Abstract, Introduction, and Discussion address the "selling points" of CoSMIC and its intended users.
- The Methods section's readability and clarity have greatly improved per the Reviewers' suggestions.
- We modified Figures 3 and 5 in the Results section to demonstrate the advantages of using CoSMIC, particularly in under-studied niches like marine sponges. The revised figures are provided below and we refer to them throughout our answers.
- As suggested, to allow general use of CoSMIC and replicating results presented in this manuscript, we have created a comprehensive code package and an elaborate tutorial outlining all aspects of CoSMIC usage.

Figures 3 and 5.

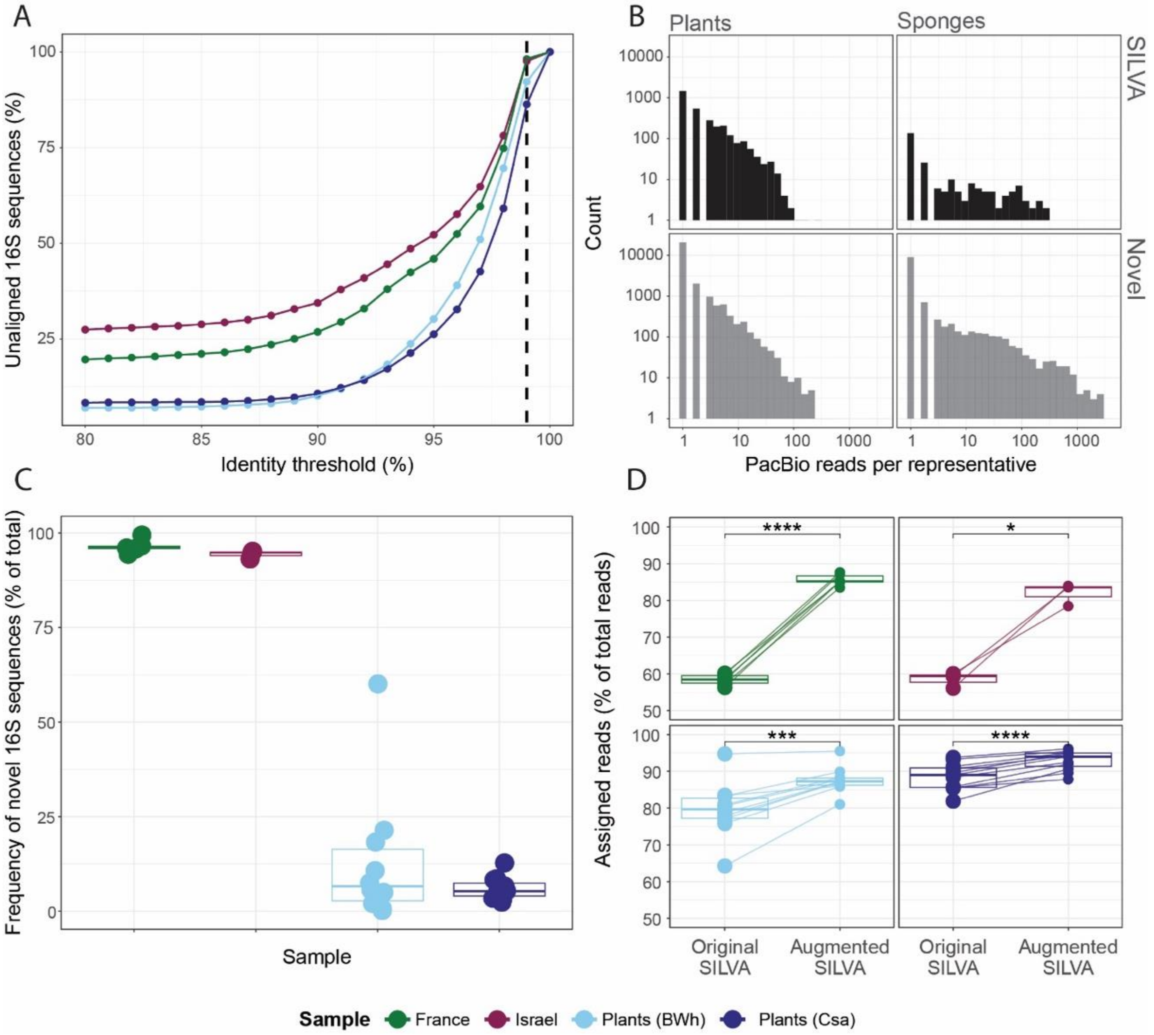

Figure 3. Enriching the database with novel full-length 16S rRNA sequences. We amplified the full-length 16S rRNA gene using pre-designed LNA primers and sequenced the resulting amplicons by PacBio long-read sequencing. Four samples were processed, each containing a pool of samples from a different habitat, i.e., plants from the Arava desert in Israel, characterized by a climate type BWh, according to Koppen climate classification; plants from the lowland region in Israel, characterized by a climate type Csa; and pools of samples from the marine sponge *Spongia officinalis* collected in the Mediterranean Sea along the coast of either France or Israel. **A.** All non-redundant PacBio sequences (Plants BWh - 14852, Plants Csa - 14589, France - 6507, Israel - 5468) were aligned to SILVA using the CD-HIT algorithm(18). The percentage of sequences that did not appear in SILVA is shown as a function of the identity threshold. The vertical dashed line corresponds to SILVA's standard threshold for calling novel full-length 16S rRNA sequences (SILVA NR99). Colors indicate different biomes sampled. **B.** Histograms of read counts per 16S rRNA sequence for the plants' pool (left panels) and the sponges' pool (right panels). The upper and lower panels show histograms of SILVA and novel sequences, respectively. An identity threshold of 99% was used to call novel sequences with respect to SILVA. **C.** Individual samples from the abovementioned pools were sequenced via short reads and analyzed by SMURF using the augmented database to determine their microbial composition (France – 6 samples, Israel – 3 samples, Plants (BWh) – 10 samples, Plants (Csa) – 10 samples). The total relative frequency assigned by SMURF to novel 16S rRNA sequences per sample (dots) and across types (columns) is shown. **D.** The fraction of reads aligned to either SILVA or augmented SILVA for each sample depicted in C. Each panel corresponds to a different sample type. To evaluate the statistical significance, a paired t-test was performed for each sample type; * P<0.05, *** P<0.001, **** P<0.0001.

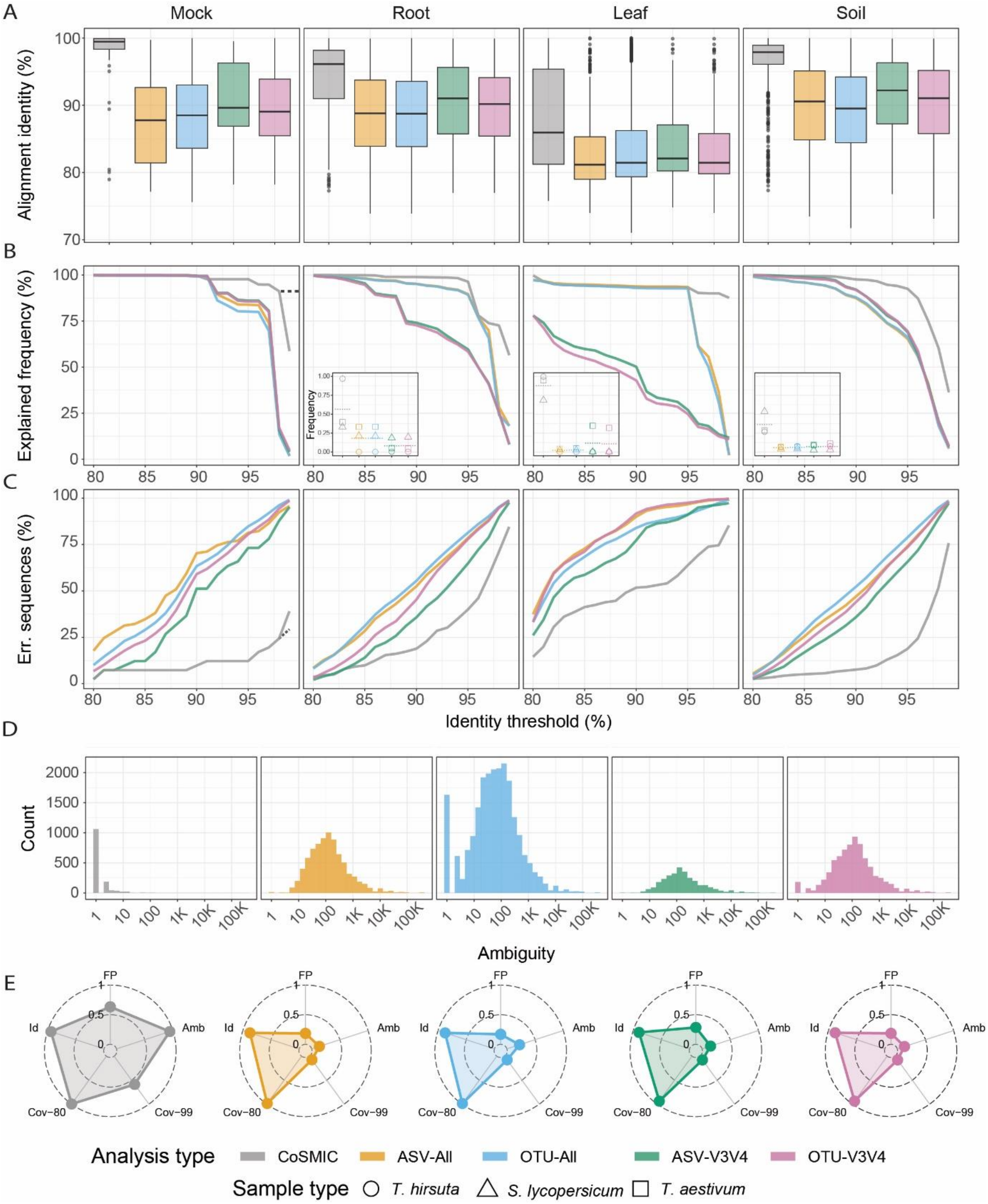

Figure 5. Experimental evaluation of standard methods vs. CoSMIC. Ten samples were sequenced - leaf, soil, and root samples of *Hymelaea hirsuta*, *Solanum lycopersicum,* and *Triticum aestivum*, and a mock mixture of 12 known species (see Methods). Each sample underwent standard shotgun metagenomic sequencing, from which we detected its full-length 16S rRNA sequences to be considered ground truth. In addition, as part of CoSMIC, we pooled extracted DNA from all ten samples, amplified by LNA primers, and sequenced via synthetic long reads (Loop Genomics) to create full-length 16S rRNA sequences that were added to the SILVA database. Standard amplicon sequencing was performed by a commercially available kit by Swift, which uses primers spanning the 16S rRNA gene to amplify several variable regions (see Methods). Analysis was then performed by CoSMIC using nine primer pairs and by a standard QIIME2 pipeline. The latter used either nine primer pairs (All) or the V3V4 region only (V3V4) and was performed using OTUs or by ASV calling. Hence, four QIIME2-based results are shown – OTU-All, OTU-V3V4, ASV-All, and ASV-V3V4. **A.** Alignment identity, i.e., the average alignment score between

detected 16S rRNA sequences and ground truth 16S rRNA genes (gt16S) detected by shotgun metagenomics. Boxplots present the distribution of alignment identities for each tissue and analysis method. Leaf, soil, and root panels correspond to an average of over three samples (one from each plant). A Mann-Whitney U test was performed for all comparisons, which resulted in very low p-values, with the highest being 0.0064. **B.** The total frequency of correctly detected gt16S by each method as a function identity threshold. The dotted horizontal line in the left panel (mock mixture) marks the results if we improve the ground truth sequences by MarkerMag. Insets present the total frequency achieved per sample at a cutoff of 99%; the dotted horizontal line is their average. The y-axis, shown for the leftmost inset, is relevant for all insets. **C.** Percentage of erroneous 16S rRNA sequences, i.e., those identified by an analysis method yet not detected by shotgun metagenomics. The percentage as a function of the identity threshold, where alignments below the threshold are considered erroneous. The dotted line marks the MarkerMag-based improvement for the mock community using CoSMIC for up to a 99% identity threshold. **D.** Histogram of the number of taxa matching an identification by each analysis type. **E.** Radar plot combining all samples over several criteria (normalized on a 0-1 scale, where large values correspond to better performance): **Id,** mean alignment identity for each analysis method as described in A; **Cov-80,** mean total ground truth frequency at 80% identity threshold as described in B; **Cov-99,** the same for 99% identity threshold; **FP,** One minus the mean false positive rate as described in C; **Amb,** mean ambiguity as described in D.

Reviewer #1

"This manuscript presents a new approach to improve characterization of microbial communities by combining long-read sequencing with multiple shorter markers, focused on 16S rRNA sequences from microbiomes. The authors focus on high-resolution microbial profiling to resolve differences of 1% sequence divergence across full-length 16S rRNA reads. The manuscript does a good job of demonstrating how shorter regions of 16S can be identical among taxa that differ in other regions; thus, any single region is likely to underestimate taxonomic diversity. The CoSMIC integration of long reads with short markers is likely to enhance the accuracy of estimates of microbial diversity; the authors note that this approach does not assess relative abundance, only presence/absence in a community.

A: We thank the Reviewer for acknowledging the advantages of applying CoSMIC. All comments are addressed below in detail.

The manuscript needs several improvements before it should be accepted for publication."

"Abstract: lines 45-48 describe the evaluation of the novel CoSMIC approach using a variety of samples, but omits the evaluation of a mock community, which is the only community for which error rates can be known. The abstract should also briefly mention these error rates for a known community, and that these rates are less than other approaches."

A: We agree that CoSMIC's error rates and performance over the mock mixture should be mentioned before describing results over "regular" samples.
We added the following text to the Abstract:

Lines 48 – 52: We first estimated CoSMIC's performance using a mock community aiming to detect all full-length 16S rRNA sequences. CoSMIC correctly identified more full-length 16S rRNA genes with significantly higher specificity than standard methods. It dramatically increased the resolution of detected sequences while avoiding the inclusion of incorrect sequences.

“Introduction: lines 83-84. Need to improve phrasing of “depend on their identity” – do you mean the identity of the particular regions? I found this sentence and the next confusing. “The issue of ambiguous assignment” – this issue is larger than what can be put into this one sentence. Do you mean assignment to OTUs? Or the assignment of a particular read to a particular SSU sequence in a database? Since this manuscript and approach do not develop a different method of matching a sequence to a database (beyond adding more SSUs to the database), I don’t think this sentence is necessary.”

A: We agree with the Reviewer’s comments. Following a comment from Reviewer 3 regarding the issue of open and closed references not being explicitly connected to this manuscript, we deleted these paragraphs and now only mention that neither OTU nor ASV solves the basic profiling problems solved by SMURF (as a precursor for CoSMIC): (a) the inherent problem of amplifying a small part of the gene; and (b) lack of comprehensiveness of databases concerning most non-human associated niches, thus hampering SMURF.

Lines 70 – 79: However, it has been widely shown that the results of such studies depend on the amplified region due to biases introduced by the specific primers applied(5,6), hence limiting the ability to harmonize findings across studies(7). Moreover, due to short read length, standard methods amplify one or two adjacent variable regions; therefore, many bacteria may share their sequence over the amplicon yet be very different along the rest of the 16S rRNA gene. Such ambiguity in assigning reads to bacteria often results in lower resolution profiling. However, current bioinformatic pipelines do not adequately address either problem, regardless of whether they use amplicon sequence variants (ASVs) or operational taxonomic units (OTUs).

“Likewise, in lines 89-90, both SMURF and the new CoSMIC rely on a database of SSU sequences; the only difference is the idea of adding new SSU sequences to the database as part of the CoSMIC workflow. I don’t understand the need for a criticism of integrating datasets using an SSU database here, when the CoSMIC workflow also relies on this same approach.”

A: Indeed, both methods depend on an SSU database. We intended to emphasize that using SMURF would yield suboptimal results when profiling unexplored niches, whose SSUs often do not appear in available databases. Hence, we did not mean to criticize using databases per se but rather highlight the problem in databases’ content in the context of non-human associated samples.
We altered the text to clarify our intention, the following text to the Introduction:

Lines 83 – 87: For such integration, SMURF relies on available databases of full-length 16S rRNA sequences (e.g., SILVA(9) or Greengenes(10)) containing millions of sequences. These databases are almost comprehensive with respect to human-associated bacteria, while other habitats may be severely underrepresented(11,12), rendering the approach suboptimal for less explored niches.

Lines 91 – 96: Before applying SMURF in a new niche, we suggest amplifying a pool of samples using generic locked nucleic acid (LNA) primers, which provide an almost universal amplification of full-length 16S rRNA sequences. Sequencing these amplicons by long-read technologies (e.g., PacBio) enriches 16S rRNA gene databases with relevant sequences, thus allowing SMURF to provide efficient and high-resolution profiling of these niches.

"Line 101, the mock mixture is not described in the abstract, but is the only way that this study can obtain an estimate of error rates across multiple approaches."

A: We edited the Abstract to address this comment (see answer above).

"Results, lines 105-112. I understand that the full Methods are provided later, but the short version here needs some additional details. It is not clear whether the CLC method used here is an open reference or a closed reference, for example. Later, in the Methods, it seems like a DADA2 open reference was used for QIIME2, but those details need to be in this brief synopsis of the methods"

A: We added clarifications about OTU picking in CLC and about DADA2 in QIIME2 analysis.
We added the following text to the relevant sections:

In Results -

Lines 115 – 118: We used Qiagen's CLC Genomics Microbiome package, which applies a closed-reference Operational Taxonomic Units (OTU) picking strategy using a similarity threshold of 97% (see Methods). The package independently analyzes each region by assigning taxonomy to OTUs detected in a region and outputs the taxonomies detected across all regions.

In Methods -

Lines 710 - 720: Standard analysis: Microbial profiling was performed using the standard Swift analysis scripts to create Amplicon Sequence Variant (ASV) or OTU tables. These tables were based on the QIIME2(20) package, where paired R1 and R2 reads were filtered and analyzed using R's DADA2(21) package to account for sequencing errors.

CoSMIC analysis: Each sample was demultiplexed to its variable regions using Cutadapt(31). In principle, since five forward primers were mixed with four reverse primers, SMURF can be applied to up to 20 specific regions. We selected nine potential amplicons (Table 3) while ignoring long amplicons (e.g., the pair V1_f and V9_r was omitted) since the number of such reads was negligible. Paired R1 and R2 reads were filtered, analyzed using R's DADA2 package to account for sequencing errors, and submitted to SMURF.

"Line 117:since this sentence is talking about beta diversity, the next sentence should start "Beta diversity was calculated by a Jaccard distance…" and not simply start with "Diversity"."

A: Corrected.

"Figures. The figures are not color-blind friendly, which became readily apparent in Figure 2. For this reviewer, in Figure 2, colors 1=4 and 2=5 for example. This comment extends to all other figures, including the supplemental figures. I suggest using colorbrewer2.org to select a suitable 6-color palette of diverging colors that are color-blind friendly. Otherwise, a substantial proportion of readers will not be able to interpret these figures."

A: We apologize for the color selection in our figures. All figures have now been revised to be color-blind friendly.

"Experimental evaluation (page 13). Why is CoSMIC only compared to the Swift framework instead of the multiple other options previously described? Why not also use an open-reference DADA2 pipeline? There needs to be some justification for the choice of approaches to compare to CoSMIC"

A: We thank the Reviewer for this comment. Our terminology needed to be clearer. In principle, following amplification by the Swift primer kit, the Swift analysis pipeline allows the user to choose whether to perform the analysis based on all regions or using a single region (e.g., V3-V4). The analysis is then performed using either an open-reference DADA2 pipeline or via a closed-reference OTU picking based on the SILVA database. Both options are applied via QIIME2.
Our original "Swift all" and "Swift V3-V4" analysis referred to an open-reference DADA2 pipeline.
For completeness, we have now added an analysis based on closed-reference OTUs.
For clarity, we have changed our terminology and call each analysis method by type:

- ASV-all: DADA2-based analysis of all regions;
- ASV V3-V4: DADA2-based analysis of V3-V4;
- OTU-all: close-reference analysis of all regions (applied database is SILVA);

OTU V3-V4: closed-reference analysis of V3-V4 (applied database is SILVA);

These results appear in the new Figure 5 and are described in the revised Results section:

Lines 351 – 354: For comparison, we provide a standard analysis provided by Swift utilizing the QIIME2(20) package to generate tables of operational taxonomic units (OTUs) or amplicon sequence variants (ASVs) utilizing DADA2(21), each based on either all amplified regions or only the widely used V3V4 amplicon.

"Page 14 and Figure 5C – the "Explained Frequency" for the Mock community drops substantially after 95% -- since this is the known community, I was surprised that the results did not include an estimate of error here. What is causing the unexplained sequences in the mock community? Is it simply error from Illumina sequencing and other laboratory methods?"

A: We thank the Reviewer for this comment, which, together with the suggestion regarding MarkerMAG, allowed us to understand this phenomenon.

First, we emphasize that Fig. 5 considers detecting all 16S rRNA sequences in each community rather than identifying the bacterial taxa. The former case is more difficult since each bacterium may harbor several copies of the 16S rRNA gene, each having (often slightly) different sequences.

The significant drop in the mock mixture in Fig. 5C (now designated Fig. 5B) occurs at an identity threshold of 98.4%. It is associated with a mismatch between an *E. coli* sequence derived from PhyloFlash and CoSMIC's *E. coli* sequence. When we aligned the Swift-based short reads (achieved at 3000-5000X depth), we found that they were in perfect match with the CoSMIC (i.e., SILVA) sequence while displaying 12 SNPs with the PhyloFlash-derived sequence. As suggested by the Reviewer in the following comment, these limitations are not restricted to PhyloFlash. To overcome this problem, we used MarkerMAG to refine the ground-truth sequences, yielding an explained frequency of 89% for identity thresholds higher than 99%.

We added the following text to the Results:

Lines 379 – 394: In three of the four cases, such reduction also seems to affect CoSMIC, yet to a lesser degree. To understand this phenomenon, we considered the mock mixture whose species composition is known. The significant drop in explained frequency occurs at an identity threshold of 98.4% and mainly originates from a mismatch between an E. coli sequence in gt16S and the detected CoSMIC sequence. Aligning Illumina short reads of the Swift pipeline to both sequences showed a perfect

match to the detected CoSMIC sequence, while the gt16S E. coli sequence contained 12 SNPs relative to the reads. Therefore, the actual value of CoSMIC's explained frequency at identity thresholds higher than 99% is at least 88%. This indicates that phyloFlash-derived 16S rRNA sequences may not be entirely accurate, demonstrating the inherent problems in achieving highly accurate assembly of 16S rRNA sequences from shotgun metagenomics. This problem is not restricted to phyloFlash and also characterizes other state-of-the-art assemblers(22,23). To alleviate this problem yet provide an estimate solely based on shotgun results, we used MarkerMAG(24) to improve the gt16S sequences (see Methods), yielding an explained frequency of 89% for identity thresholds higher than 99% (Fig. 5B left panel, grey dashed line).

Lines 443 - 446: The total frequency of correctly detected gt16S by each method as a function identity threshold. The dotted horizontal line in the left panel (mock mixture) marks the results if we prune the ground truth sequences by MarkerMag. Insets present the total frequency achieved per sample at a cutoff of 99%; the dotted horizontal line is their average.

"Figure 5D – the "Erroneous SSUs" are those not detected by shotgun metagenomics. In this case, I was very surprised that the mock community rate of Erroneous SSUs was over 40% ! However, there are known limitations in using Illumina-platform sequencing to detect ribosomal genes in shotgun metagenomics. The authors should an include a discussion of this point and potentially use the MarkerMAG software to increase the recovery of 16S sequences from their metagenomic libraries: https://doi.org/10.1093/bioinformatics/btac398 "

A: We thank the Reviewer for this comment. We added the post-MarkerMAG percentage of erroneous sequences for the mock mixture (Fig. 5C dashed line in left panel). While MarkerMAG decreases the percentage of erroneous sequences by only 9.5%, we note that it allows explaining 89% of the mock mixture community at an identity level of 99%. Hence, erroneous sequences correspond to only ~11% of the population. Moreover, the high percentage of erroneous sequences for the mock at high identity thresholds corresponds to merely 12 sequences, as shown in the new Fig. S14 (provided below).
Such errors may also be another manifestation of the limitations of shotgun-based ground truth. Although more than 60 million reads were allocated to shotgun sequencing of the mock mixture, only ~88,000 were assigned to 16S rRNA sequences. Hence, the ground truth may be missing some sequences, and the erroneous sequences at 99% identity may be lower than 12.

We added the following text to the Results:

Lines 397 – 413: Erroneous 16S rRNA sequences - The percentage of erroneous sequences, i.e., those reported yet not present in the ground truth sample, is

significantly lower for CoSMIC than the other methods (Fig. 5C). Moreover, in three of the four cases, the number of erroneous sequences using standard methods grows linearly with the identity threshold, while CoSMIC displays an increase starting from a threshold of 95%. To understand this phenomenon in the case of the mock mixture, we used the MarkerMAG-improved sequences as ground truth, yielding a reduction of 9.5% (Fig. 5C left panel, grey dashed line). Although the percentage of erroneous sequences at a high identity threshold is still significant, we note that since MarkerMAG-based sequences explain 89% of the mock mixture sequences at an identity threshold of 99%, the erroneous sequences correspond to only ~11% of the population. Moreover, the absolute number of these erroneous sequences is merely 12 (Fig. S14). In addition, these errors may also be another manifestation of the limitations of shotgun-based ground truth. Although more than 60 million reads were allocated to shotgun sequencing of the mock mixture (Fig. S15), only ~88,000 were assigned to 16S rRNA sequences. Hence, the ground truth may be missing some sequences, and the actual erroneous sequences at 99% identity may be lower than 12.

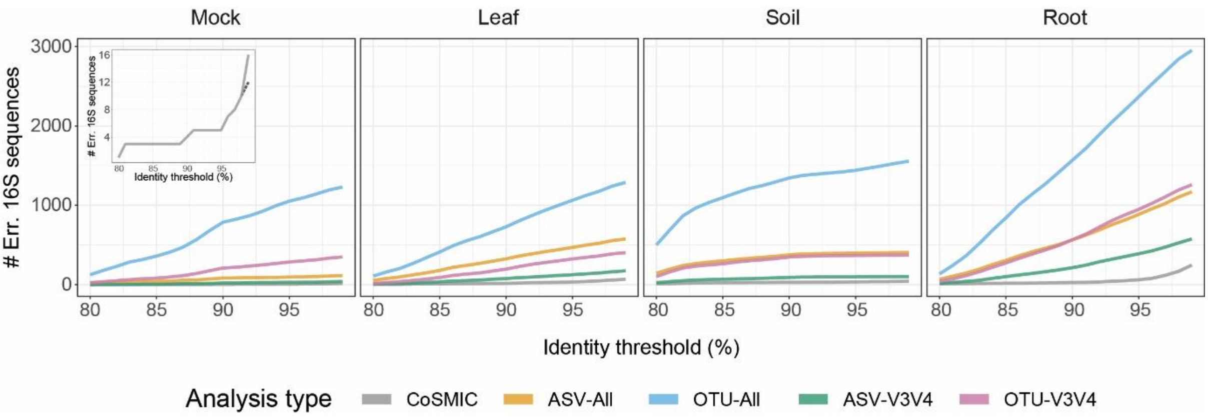

Figure S14. The absolute number of erroneous 16S rRNA gene sequences. A supplementary figure to Fig. 5C shows the number of erroneous 16S rRNA gene sequences detected by each method for each sample type as a function of the identity threshold. Erroneous sequences are those that were identified by each method yet were not detected by shotgun metagenomics for each identity threshold. The inset for the Mock mixture (left panel) zooms into CoSMIC's results. The dotted line corresponds to CoSMIC's results for up to a 99% identity threshold when improving the ground truth via MarkerMag.

"Discussion: The discussion does not place these findings into a context of cost-effectiveness in terms of money, time, personnel, and benefit to the researchers. If the full-length SSUs are optimal, why not stop at the first step in the workflow, and spend more effort on generating full-length SSUs from each sample? What is the relative cost of moving from one to four (or more) SSU regional markers? Is the cost simply 4x the money and time? If so, how much more accuracy is gained? A more complete discussion needs to place the current recommendations to use the CoSMIC framework into a discussion of what researchers can gain, and at what cost."

A: We thank the Reviewer for these comments. Applying a single primer pair yields lower diversity due to amplification biases and may also provide lower resolution. Both problems are alleviated by using all available primer pairs via CoSMIC.
Overall costs per sample in CoSMIC (including all expenses - from DNA extraction to those related to short- and long-read sequencing) range between $25 to $30, depending on the number of primer pairs applied. Hence, overall costs only marginally depend on the number of primer pairs since the expensive stages are DNA extraction and other sample preparation steps, which do not depend on the number of applied primer pairs. Moreover, amplification is often applied in multiplex; thus, sample preparation and labor may be comparable to using a single primer pair. However, large-scale projects may benefit from a 10-15% reduction in costs associated with the number of applied primer pairs; therefore, we advise researchers to estimate the minimal subset of primer pairs that provide adequate results for their samples (e.g., Fig. 2).
Regarding using PacBio over individual samples (instead of over pools of samples), while PacBio offers high accuracy, its costs become prohibitive for large projects. For example, the estimated cost for PacBio sequencing is $300 per sample for up to tens of samples, and even higher costs for a larger number of samples due to the price of additional indices. Hence, CoSMIC provides a means of circumventing such high costs while providing analogous results.

To convey these points, we added the following text to the Discussion:

Lines 489 – 499: Overall costs per sample in CoSMIC (including all expenses - from DNA extraction to those related to short- and long-read sequencing) currently range between $25 to $30, depending on the number of primer pairs applied. Hence, overall costs only marginally depend on the number of primer pairs since the expensive stages are DNA extraction and other sample preparation steps, which do not depend on the number of applied primer pairs. Also, amplification is often applied in multiplex; hence, sample preparation and labor may be comparable to using a single primer pair. Moreover, large-scale projects may benefit from a 10-15% reduction in costs associated with the number of applied primer pairs. Therefore, we advise researchers to estimate the minimal subset of primer pairs that provide adequate results for their samples (e.g., Fig. 2).

The straightforward approach of using our generic LNA primers and applying PacBio long-read sequencing to individual samples rather than pools of samples is possible and would achieve equivalent results to CoSMIC. However, its costs become prohibitive for large projects. For example, the estimated cost for PacBio sequencing is about $300 per sample for tens of samples and even higher costs for larger cohorts due to the price of additional indices. Hence, CoSMIC provides a means of circumventing such high costs while providing analogous results.

"The Discussion section also misses a major opportunity to compare error rates among the different approaches tested with the mock community. Adding such a comparison would greatly improve the manuscript."

A: We agree and have added a discussion regarding the mock community errors:

Lines 480 – 488: To our surprise, CoSMIC also outperformed standard methods when profiling a mock community of known species. The average alignment score between 16S rRNA sequences detected by CoSMIC and the ground truth sequences was 97.2% (Fig. 5A left panel), while the score of standard methods was lower by ~8%. This corresponds to a ~120bp difference along the gene, thus potentially leading to a lower resolution. Also observed was an overestimation of the diversity of 16S rRNA sequences over the mock mixture, evident in both ASV or OTU-based standard analysis (Fig. 5C left panel). Hence, CoSMIC provides a more robust estimation of this important ecological property.

"Technical comments -"

"Figure S10 and several others: the X-axis labels are difficult to parse. There needs to at least be a statement in the figure caption to refer to a table to understand what these abbreviations mean.
Figure S11: again, the x-axis labels are not explained in the figure caption.
Figure S13: again, the x-axis labels are not explained in the figure caption.
Figure S14: same comment.
Table S1: need to indicate the number of individuals comprising each pool of multiple samples."

A: We have changed all figures according to the comments mentioned above

Reviewer #2

Reviewer #2 (Comments to the Author):
"X et al present COSMiC, an extension of their previously published subunit scaffolding algorithm to extend it to novel environments. The authors first demonstrate that individual primer pairs lead to a decrease in community-based resolution compared to the use fo multiple regions, which are solved with their SMURF algorithm. They then raise the issue that a publicly available database may have a major disadvantage for a specific environment, and propose supplementation with novel diversity introduced via long read sequencing. They evaluate their COSMiC pipeline comparing an original and augmented SILVA database, basing the result on a metagenomic profiling approach. The authors conclude that this pipeline is a major improvement over current technologies for 16S identification.

I am unable to evaluate COSMiC as a method, since the provided code is incomplete and insufficiently annotated. I do not know the environmental requirements, how to install the software, nor could I run a tutorial. The GitHub was somewhat unenlightening on how to do the core method described in the paper. I don't have enough information to run what's already been done, let alone apply this technique to my own data. With that limitation, I don't think this paper can rightly be called a methodological paper because the computation is a key component of the method, and I'm not sure the population I can find sufficiently represents a new algorithm."

A: We thank the Reviewer for this comment. Indeed, computation is a key component of the method. Therefore, the code should be readily provided and well documented. We have made a significant effort to improve the accessibility and utility of the CoSMIC pipeline. More specifically, we have created a new code package that includes all steps in the CoSMIC pipeline:

- Preparing long-read 16S rRNA gene sequences to be augmented to an existing database.
- Adding novel full-length 16S rRNA sequences to the database.
- Preparing the augmented database in a SMURF-like manner, i.e., considering a specific set of primer pairs.
- Denoising and demultiplexing of Illumina short reads from the samples to be profiled.
- Running SMURF based on the augmented database and using denoised reads.
- Combinatorial comparison of primer sets - used to evaluate profiling quality of specific subsets out of the full set of primer pairs.

These steps have been compiled in a comprehensive step-by-step tutorial that includes data requirements and installation prerequisites.

We hope the current tutorial and code package will allow widespread use of CoSMIC.
We added the following text to the Methods section to direct potential users to our site. A Zenodo link will be created upon approval by the Reviewers and Editor.

Lines 782 – 798: The CoSMIC software package

The software package provides a comprehensive set of scripts facilitating all steps of CoSMIC. The package includes scripts augmenting an existing database by full-length 16S rRNA sequences obtained from long-read sequencing (e.g., PacBio). It then provides code that formats the augmented database in a SMURF-like format per the specific primers' set to be used in the short-read sequencing experiments. The package includes scripts for denoising and demultiplexing paired-end Illumina short reads using the DADA2 algorithm and running SMURF. The package supports SMURF profiling based on any subset of the original set of primers.

"That said, even if the method satisfied the basic criteria of installable and sufficiently documented to be runnable on tutorial data, additional clarification is needed about who the intended userbase/audience would be. This seems like an expensive approach, which requires substantial work to design an appropriate LNA for a region; multiple kinds of sequencing; and likely substantial computation. Given those requirements, it seems like the method needs something to set it apart from more common methods (i.e. metagenomic and long read sequencing). Is there a cost advantage? A time advantage? A bias advantage? What is the selling point for this over other techniques?"

A: We thank the Reviewer for emphasizing the need to clarify all these points. We address them below, where titles refer to each Reviewer's comment.

"*additional clarification is needed about who the intended userbase/audience would be*"

Response: When designing CoSMIC, we sought to provide a SMURF-like high-resolution microbial profiling of any non-human associated niche, i.e., whenever current full-length 16S rRNA databases fail to capture the niches' bacteria. We therefore hope that CoSMIC will be adopted by researchers seeking efficient and accurate large-scale profiling of non-human niches. For example, the revised Figure 3 shows that, on average, 95% of the overall frequencies of the detected communities in our sponge samples correspond to novel full-length 16S rRNA sequences added to SILVA by PacBio LNA-based sequences. Hence, CoSMIC provides more accurate and high-resolution profiling in this case.

To clarify the intended "audience" the following text was added to the Discussion:

Lines 464 – 468: Applying CoSMIC is more important in less explored environments, e.g., marine sponges vs. soil samples, where close to 100% of the reads align to novel 16S rRNA gene sequences detected by CoSMIC (Fig 3D). Therefore, we hope CoSMIC will be adopted by researchers seeking efficient and accurate large-scale profiling of less explored niches.

“This seems like an expensive approach, which requires substantial work to design an appropriate LNA for a region; multiple kinds of sequencing; and likely substantial computation.”

To clarify the issues of cost and computation, the following text was added to the Discussion:

Lines 478 – 488: Overall costs per sample in CoSMIC (including all expenses - from DNA extraction to those related to short- and long-read sequencing) currently range between $25 to $30, depending on the number of primer pairs applied. Hence, overall costs only marginally depend on the number of primer pairs since the expensive stages are DNA extraction and other sample preparation steps, which do not depend on the number of applied primer pairs. Also, amplification is often applied in multiplex; hence, sample preparation and labor may be comparable to using a single primer pair. Moreover, large-scale projects may benefit from a 10-15% reduction in costs associated with the number of applied primer pairs. Therefore, we advise researchers to estimate the minimal subset of primer pairs that provide adequate results for their samples (e.g., Fig. 2).

Response regarding LNA: It is important to clarify that LNA primers are generic and do not need to be customized per study or experiment.
To explain this point, the following text from the Results section, “Enriching the database using long-read sequencing,” was edited:

Lines 231 - 234: We, therefore, propose to pool samples from the relevant niche, apply PCR amplification using the pre-designed LNA primers, and sequence by long reads. To account for potential chimeras, the resulting reads undergo several quality assurance steps and are clustered at 99% identity to remove redundancy.

Response regarding multiple kinds of sequencing and likely substantial computation: Utilizing CoSMIC does require long and short-read sequencing. However, we note that long-reads are only scarcely used throughout a project’s lifetime, i.e., on its onset, and then only if there is an indication that another database augmentation is required. Hence, from the perspective of a large-scale project, the main sequencing workhorse is the short-read technology. We encourage researchers to invest in long-read sequencing, as illustrated in Fig. 3, since it facilitates higher

resolution for their project and serves the entire community by potentially enriching 16S rRNA sequence databases.

As for computing requirements, we often use a single Linux machine for CoSMIC or SMURF reconstructions. The intensive part of this process is creating the database in a standard SMURF format (see the script `run_create_SMURF_db.sh`), which should preferably allocate one core per amplified region (e.g., 9 cores for the case of the Swift primer set) and takes several hours to complete.
It is not a limiting factor because it is performed only once, i.e., when: (a) augmenting the full-length 16S rRNA database with new sequences; and (b) using a new set of primers. (e.g., the script has to be run once for the Qiagen set of six primer pairs, even if subsequent SMURF reconstruction is performed using different subsets).

The common step of sample reconstruction may be performed using a single core, taking a few minutes per sample. It can also be parallelized to split the set of samples across available cores.

The supplied package was optimized for efficiency, requiring a 20-core machine with 16GB of RAM per core, a specification within reach for most academic settings.
With adjustments, our processes can run on standard PCs, albeit more slowly, making our pipeline accessible and practical for a wide range of research environments without exceptional computational resources.

The following text was added to the Methods:

Lines 803 - 810: Hardware requirements

The CoSMIC package was optimized for efficiency, requiring a 20-core machine with 16GB of RAM per core, a specification within reach for most academic settings. With adjustments, our processes can run on standard PCs, albeit more slowly, making our pipeline accessible and practical for a wide range of research environments without the need for exceptional computational resources.
Documentation for code and processing requirements is provided at the GitHub repository.

Response regarding cost advantages, time advantages, a bias advantage, and the selling point for CoSMIC over other techniques:

“Selling points” and cost advantages: The main “selling point” for CoSMIC is the ability to perform 16S rRNA profiling of non-human-associated niches while achieving a de facto full-length 16S rRNA resolution via regular short-read sequencing, i.e., at a fraction of the cost of applying PacBio for this task (see costs estimated in our reply to Reviewer 1 and the abovementioned text). Second, CoSMIC provides flexibility in

primer selection, allowing researchers to evaluate the utility of each primer pair and select the minimal set that achieves sufficient resolution.

The “selling point” was mentioned in the Discussion (see the abovementioned text regarding the “audience”) and in the Abstract:

Lines 57 - 58: CoSMIC is well-suited for profiling unexplored habitats, offering full-length 16S rRNA resolution at a fraction of the cost of per-sample long-read sequencing.

And Introduction:

Lines 101 - 105: CoSMIC is best suited for profiling novel ecological niches. It provides de facto full-length 16S rRNA resolution at about 10% of the cost of per-sample PacBio sequencing. This cost-effectiveness, combined with CoSMIC's flexibility in primer selection, allows researchers to optimize their experimental designs for both scope and budget.

Sample preparation time: Applying CoSMIC does not offer a time advantage, yet we note that sample preparation does not scale with the number of amplified regions due to the calibrated multiplexed reaction (the former text refers to this point also).

Reduced primer and amplification bias: Primer bias is less crucial in the LNA-PacBio amplification and sequencing step since the sole objective is to provide full-length sequences, while incorrect abundances due to PCR biases are less important. As for the SMURF part of CoSMIC, since multiple primer pairs are applied, primer biases are “averaged” across the different pairs and thus potentially attenuated. We note that, in this manuscript, we have not emphasized the issue of detecting correct abundances. As mentioned in the original Discussion, we are now calibrating an augmentation of CoSMIC, which applies unique molecular identifiers for improved abundance estimates.

“A few other issues to consider:”

“1. The discussion of older table construction techniques in the introduction is incorrect in several places. Specifically, open reference OTU clustering is incorrectly defined, and the section on denoising requires review.”

A: We agree with the Reviewer. For conciseness, and per a remark by Reviewer 3, we deleted the paragraphs regarding OTU and ASV and now focus the Introduction on the two issues that drive CoSMIC: (a) the inherent problem of amplifying a small part of the gene, i.e., the problem solved by SMURF; and (b) lack of comprehensiveness of databases concerning most non-human associated niches, thus hampering SMURF.

"2. The multi-region section would benefit from a discussion of database coverage. For instance, does the V13 region fail to add information because all the reference sequences there are already covered by the V34 region?"

A: Two phenomena are associated with adding a region. First, each region may detect bacteria that are not amplified in other regions (as shown in Fig. 1). The second phenomenon is related to a reduction in ambiguity. As stated in the Reviewer's example, if all bacteria detected by V34 have distinct sequences over V13 then indeed, V13 is "redundant" and would not reduce the ambiguity, i.e., the number of reference sequences which are "indistinguishable" via V34+V12. We note that this is often not the case, and there is a significant reduction in ambiguity when comparing results from a single region to those provided by two regions (Fig. 1A).

"3. For the distance between region, how does this compare to the same sample and same region sequenced twice? Alternatively: how much of the distance is due to stochasticity in PCR and processing; how much is due to primer bias; and how much is due to database coverage, if you can break those apart?"

A: The samples in Fig. 2 were processed once, i.e., we did not repeat DNA extraction, sample preparation, or sequencing. Hence, we cannot estimate the "stability" of these results under technical or biological repeats. However, each dot in Fig. 2 corresponds to an average of 24 samples, thus supporting its "stability".
The variability shown in Fig. 2 primarily reflects primer bias, database coverage, and SMURF's ability to extract information from different sets of regions.
Although we cannot break these apart, Fig. 2 demonstrates that the same primer combination may have a different effect across tissues. Hence, researchers should select their optimal set by evaluating their performance over the relevant samples, similar to the 24 samples in this case.

"4. The databases used in various steps in the analysis need to be clarified. I know SMURF originally used a filtered, manually curated Greengenes 13_5 or 13_8. Given the advancement in databases in the last decade and multiple commentaries on database quality, has the issue COSMiC is trying to solve already been solved with a newer database?
Could the issues be solved through a more precise de-replication of the existing database, or does the comparison to a subset really provide novel diversity?"

A: This study uses SILVA, which is, as far as we know, the most comprehensive and updated database available (Greengenes was not used here). Yet, we show that 95% and 85% of PacBio-detected full-length 16S rRNA sequences are novel according to the SILVA standards for sponges and plants, respectively (Fig. 3A). The difference in sequence between PacBio-detected sequences and SILVA is so large that a more precise de-replication would not have bridged this gap.

We also show that proactively enriching the database with niche-specific full-length sequences is required for implementing SMURF in these cases (see the total abundance of non-SILVA sequences in profiled sponge samples in Fig. 3C, and the percentage of assigned reads in Fig. 3D).

These points were added to the Results section describing Fig 3:

Lines 234 – 238: A representative sequence from each cluster is aligned to the SILVA NR99 database(9), and sequences whose alignment to SILVA is lower than 99% are considered novel (for a detailed description of the abovementioned steps, see Methods). We refer to the database containing SILVA and the novel sequences as “augmented SILVA”.

Lines 247 – 254: Figure 3A presents the percentage of novel sequences as a function of the identity threshold when aligning to SILVA. Sponges seem to have significantly higher percentages of novel sequences(17), irrespective of identity threshold. Using the SILVA definition of a novel full-length 16S rRNA sequence, i.e., a threshold of 99% identity (vertical dashed line in Fig. 3A), more than 95% of detected sequences were novel for samples of sponges and about 85% for samples taken from plants. These novel sequences include many whose relative abundance in the pool is high, especially in sponges.

Regarding the de-replication of the existing database, we show in the now altered Fig 3 that for a vast majority of the relevant 16S rRNA sequences, the closest relative is Bigger than a 20% difference (Fig 3A), so even if the problem of ambiguity could be slightly alleviated by de replication many of the actual participating species wouldn’t be aptly characterized by current databases. These species are not scarce and could be highly prevalent (Fig. 3B), and have close to 100% of the reads attributed to them in the case of sponges (Fig 3C), so they hold important biological meaning.

“5. There’s a promise of phylogeny somewhere in the analysis, but I don’t see any phylogenetic anything here?”

A: We have deleted phrases regarding “phylogenetic resolution”. We meant to write that we provide the highest possible sequence resolution available by the 16S rRNA gene. The text was changed accordingly.

“6. The results section needs more time breaking things down and describing what I should be getting from the figures/summary”

A: We agree with the Reviewer. The Results sections, especially those referring to Figs. 3 and 5, have been elaborated. We added panels, and the relevant text has been expanded to allow easier understanding.

“*7. The “samples” and “processing” section of the methods section could perhaps be simplified and refined. I found the structure of sample, then processing confusing. It might be more clear if, for each processing pipeline, you also presented the samples and bioinformatics”*

A: We agree with the Reviewer’s comment. The revised Methods section was reordered - we present samples, bioinformatics, and other steps per processing pipeline.

“*8. The references in the methods section are incorrectly formatted and do not align with the existing methods section”*

A: Corrected.

“*9. Data should be deposited in a repository. A personal or institutional dropbox runs the risk of link rot. I would also recommend that once there is a functional GitHub method it be deposited in Zenodo to give a persistent identifier.”*

A: We have now created a more comprehensive software package and deposited it to GitHub (https://github.com/NoamShental/CoSMIC). Once the Reviewers and Editors approve it, we will deposit it into Zenodo.

Reviewer #3:

"Knafo and colleagues present CoSMIC, a novel approach for 16S rRNA gene-based taxonomic profiling of microbial communities. The approach combines a previously established primer set for multi-amplicon sequencing with the SMURF method, which the group of the corresponding author developed recently.
The authors explain the methodology and benchmark it against other 16S rRNA gene amplicon methods."

"Overall, the manuscript reads like the instructions to apply the author's method. Given that the method is quite elaborate, this is partially justified but obscures (at least to me) the novelty of the manuscript. The method sounds intriguing but the novelty over e.g. SMURF needs to be worked out better and communicated explicitly."

A: We thank the Reviewer for this comment and agree that the delta between SMURF and CoSMIC should have been phrased as clearly as possible. SMURF and CoSMIC require a database of full-length 16S rRNA sequences to solve the optimization problem and reach a solution that combines all regions. However, SMURF implicitly assumed that the database is comprehensive, which is correct only for human-associated niches. CoSMIC aims to bridge this gap cost-effectively by introducing a preliminary step of proactively enriching the database via long-read sequencing over pools of samples.

The following text was added:

In the Introduction:

Lines 80 - 91: To overcome these problems, we have previously presented the Short MUlti-Region Framework (SMURF) approach(8). In SMURF, each sample is amplified by any number of primer pairs, and reads from different regions are computationally combined to provide coherent, high-resolution microbial profiling. For such integration, SMURF relies on available databases of full-length 16S rRNA sequences (e.g., SILVA(9) or Greengenes(10)) containing millions of sequences. These databases are almost comprehensive with respect to human-associated bacteria, while other habitats may be severely underrepresented(11,12), rendering the approach suboptimal for less explored niches.

Here, we present Comprehensive 16S rRNA gene Mapping and Identification of Communities (CoSMIC), which builds on SMURF to provide a framework for high-resolution profiling of novel niches.

In the Discussion:

Lines 466 - 471: To do so, SMURF requires a comprehensive database of full-length 16S rRNA sequences. Yet, such comprehensiveness holds mainly for human-

associated samples, and SMURF's performance is sub-optimal for other niches (e.g., Fig. 3C-D). CoSMIC aims to bridge this gap in a cost-effective way by introducing a preliminary step of proactively enriching the database with niche-relevant full-length 16S rRNA sequences.

To verify that CoSMIC is indeed necessary, we added two panels to Figure 3 (see the figure as part of our response to Reviewer 2), displaying (a) the total abundance of all "new" full-length 16S rRNA sequences across samples. For example, in the case of sponges, "new" sequences correspond to more than 95% of the total abundance across samples; and (b) an increase in the fraction of assigned reads after augmenting the database, i.e., showing that CoSMIC manages to "explain" more of the underlying community.

"The establishment of the new sequence database and the benchmarking of the methodologies both seem off. I will add specific comments below and mention the specific figures and paragraphs that I find to have issues.

Across the manuscript, many statements are made without literature support. There are too many to list individually here; hence I ask the authors to carefully check each statement made across the manuscript and back it up by literature references."

A: We thank the Reviewer for this comment. Statements have been backed up by literature references.

"The shorthand SSU stands for small ribosomal subunit as mentioned in the text. Yet, in the text is it used to mean small ribosomal subunit gene sequence. Since the shorthand SSU has a distinct meaning within the existing literature, I ask the authors to rework their manuscript to include a non-ambiguous shorthand for 16S rRNA gene sequences. It also seems to be used ambiguously for sequence and some kind of broader stricter OTU representative representing highly similar sequences. This needs to be clarified throughout the manuscript."

A: We agree that SSU might be confusing terminology. We have changed the term to "full-length 16S rRNA sequence" throughout the manuscript.

"A lot of space is used on open and closed reference sets, but this is not connected explicitly to the rest of the manuscript. Also, the open-reference explanation seems very short and misses important points about that strategy (while closed reference is discussed in detail, so there is space)"

A: We agree with the Reviewer. We deleted the Introduction paragraphs regarding OTU (closed/open reference) and ASV since they are not explicitly connected to the rest of the manuscript. The Introduction focuses on the two issues that drive CoSMIC (for the current Introduction text, see the answer to Reviewer 2).

“Line 110: more information about the OTU clustering is needed + this needs more context, for example, basic information such as the clustering threshold. What is known about differences in the speed of evolution between the different V regions?”

A: These details have been explained in the Results and Methods section, as also requested by Reviewer 1.

The following text was added to the Results:

Lines 113 - 119: Profiling was conducted using the commercial Qiagen QIAseq 16S/ITS Region Panel(13), which amplifies six regions along the gene (Fig. 1A), resulting in 200K-600K reads per sample (Fig. S1). We used Qiagen’s CLC Genomics Microbiome package, which applies a closed-reference Operational Taxonomic Units (OTU) picking strategy using a similarity threshold of 97% (see Methods). The package independently analyzes each region by assigning taxonomy to OTUs detected in a region and outputs the taxonomies detected across all regions.

And Methods:

Lines 586 – 592: The resulting FASTQ files were analyzed using the QIAGEN CLC Genomics Workbench software with the CLC Microbial Genomics module, which performs closed-reference Operational Taxonomic Unit (OTU) analysis. More specifically, the software preprocesses the raw sequencing data, aligns the reads against a reference database (Greengenes(10) clustered at 99%), and clusters the sequences into OTUs based on a similarity threshold of 97%. It then assigns taxonomy to each OTU and generates an OTU table containing the frequency of each OTU in each sample.

“Line 168: The ambiguity will always decrease when using longer sequences, this should be mentioned”

A: We agree. Amplifying additional regions will always reduce the ambiguity compared to the baseline.

The following text was added to the Introduction:

Lines 173 - 176: Our assessment of each region's contribution was based on three parameters: *(a)* ambiguity, i.e., the average group size detected by SMURF. The lower the ambiguity, the higher the resolution, as could be expected from the added regions;

“Line 169: The use of all detected "SSU" (as said, please change this term) as a gold standard does not seem justified well. False positives due to sequencing chimeras etc can play a role here and should be accounted for.”

A: We apologize for being unclear. We use the 6 regions as a benchmark since this is the best solution SMURF can reach in this case. Applying SMURF to any subset of these regions would create “inferior” results. The only question is how quickly results deteriorate and whether there is a trade-off between the quality of results and overall costs. Hence, it might be possible to have satisfactory results using a specific subset of primers. Whenever amplification is performed in multiplex, the main cost of adding a region corresponds to the costs of sequencing reads, which, although relatively cheap, may be important in large-scale surveys.

To justify using all regions as a benchmark, the following text was added to the Introduction:

Lines 176 - 179: relative detection, representing the fraction of identified 16S rRNA sequences out of those identified when all six regions are used (Results based on all six regions are the best possible in this case; hence, all subsets are compared to them;

As for chimeras, we wanted to clarify that SMURF is robust to detecting chimeric sequences for two reasons:
(a) SMURF can be considered a “closed-reference” approach since it outputs only full-length 16S rRNA sequences that are part of the database being applied. Hence, if the database itself is chimera-free, the output of SMURF contains “legitimate” 16S rRNA sequences.
(b) Even if the database of full-length 16S rRNA sequences does, for some reason, contain chimeric sequences, SMURF would not select them as part of its reconstruction. The reason is that such sequences would be inconsistent with short reads from the different regions.
See also our response below regarding potential chimeric sequences resulting from PacBio sequencing and the relevant text in the manuscript.

“Line 172: This statement should be revisited. It seems obvious that the results become more similar when the underlying data becomes more similar. That is: using all regions as the gold standard implies that using many regions instead of just one will yield more similar results. This does not really imply that one is better than the other though.”

A: Our statement is that not all combinations of a given number of regions provide equal results, as can be observed by the dispersion of results, e.g., Fig. 2A for pairs of regions. Moreover, the performance of each specific set depends on the particular niche. We believe that the following text from the Results section conveys this point:

Lines 198-201: For example, combining three primer pairs, V1V2, V2V3, and V4V5, yields better results in soil samples than some sets of four primers (Fig. 2D, red cross vs. all other pink circles). However, the same combination performs poorly for root (Fig. 2D-E, red cross) and leaf (Fig. S9, red cross).

“Line 177: This sentence requires more context.”

A: The sentence was revised in the Results section:

Lines 186 - 188: Since sequences belonging to the same group differ by un-sequenced regions, including each additional region removed sequences that were inaccurately declared by the smaller number of regions.

“Line 222: This procedure seems prone to chimeric amplifications. An analysis showing possible chimeras would be a good addition to the manuscript”

A: We thank the Reviewer for this comment. Two chimera detection mechanisms were applied. First, PacBio uses the SMRTseq process, which eliminates chimeric reads as a QA step during its analysis when two SMRTbell adaptors are sequenced. Second, we used “vsearch uchime_denovo” to detect chimeric reads (none were found). We advise researchers using CoSMIC to apply these two steps before uploading novel sequences to public repositories.

Moreover, we emphasize that chimeric 16S rRNA sequences, i.e., incorrect sequences, do not affect the output of SMURF. SMURF cannot select such sequences as part of its reconstruction results since they cannot correspond to the set of sequenced short reads across regions. Hence, even if chimeric PacBio reads enter our list, for some unknown reason, their only effect would be increasing the database size.

The following text was added to the Results, Discussion, and Methods:

In the Results:
Lines 233 - 234: To account for potential chimeras, the resulting reads undergo several quality assurance steps and are clustered at 99% identity to remove redundancy.

In the Discussion:
Lines 524 - 530: Also, chimeric reads that end up in an augmented database cannot be part of CoSMIC’s reconstructions since there will not be a set of short reads that supports such an erroneous sequence. However, as performed here, we advise researchers to apply chimera-detection (e.g., intrinsic algorithms by PacBio, uchime_denovo, etc.) prior to adding sequences to the ad-hoc database to keep the database “clear” of irrelevant sequences.

In the Methods:
Lines 662 - 665: An initial analysis step of demultiplexing and filtering was performed using the SMRT(28) analysis software. The software also detects and discards chimeric reads (reads are eliminated if two SMRTbell adaptors are sequenced).

Lines 674 - 679: **Filtering potential chimeras**:
Although the SMRT analysis software already filters out potential chimeric reads, and to support chimera detection for Loop Genomics reads, the uchime_denovo of VSEARCH(30) was applied to detect and remove chimeric sequences. No chimeric reads were found among full-length 16S rRNA sequences that were to be “augmented” to our database.

“Line 248 and Figure 2: The linear growth of the number of SSUs and the large number of singletons imply either severe undersampling or the generation of chimeric sequences. I ask the authors to look into this in detail”

A: The linear growth is consistent with the inflation of singletons (former Figure 3B and the histograms in new Fig. 3B). As in our reply to a former comment, we claim these are not chimeric sequences. As noted by the Reviewer, we believe we are undersampling the population, yet it mainly affects low-abundance species (as evident from new Fig. 3C).

“Figure 4: A) Alignment identity: The explanation in the methods is unclear and thus the shown results seem off. 16S rRNA gene sequence alignment of below 90% indicates that the used database is missing many important taxa. As the CoSMIC results are near perfect (for much longer sequences nonetheless), the database should include all important taxa. I cannot understand what was done from the methods, so this needs to be explained in more detail and the discrepancy needs to be investigated.”

A: The Methods section regarding Fig. 5 was rewritten (provided below) to clarify the analyses.
Regarding the discrepancy between CoSMIC results and the other methods - CoSMIC uses the “augmented” database while other methods use SILVA. Hence, some sequences are unavailable for standard closed-reference methods, resulting in lower identity scores. Second, CoSMIC utilizes all sequenced regions, thus providing better results than methods based on V3V4.

Methods section “Calculating alignment identity, explained frequency, erroneous 16S rRNA sequences, and ambiguity in Figure 5”:

Lines 766 - 791: The output of SMURF is a set of groups, where each group includes 16S rRNA genes whose sequence is identical over the amplified regions yet varies

over the rest of the gene. An analogous definition of a group for standard analysis was created based on the SILVA database, in which we extracted all relevant 16S rRNA gene sequences that share the same taxonomy as set by the QIIME2 pipeline. For example, if QIIME2 provides a genus-level taxonomy for a certain ASV or OTU, then its “group” comprises all SILVA sequences of this genus. This definition is equivalent to SMURF’s groups as all 16S rRNA sequences sharing the same taxonomy are indistinguishable based on the reads provided.
Groups of QIIME2 or CoSMIC were used for the comparisons below:

Ambiguity (Fig. 5D): Ambiguity is the number of full-length sequences in a group. Fig. 5D displays a histogram of group sizes for both CoSMIC and QIIME2-based analyses.

The other measures in Fig. 5 required processing the groups. The sequences in each group were clustered using CD-HIT, and the most frequent sequence was selected as the group’s representative.

Alignment identity (Fig 5A): Each representative sequence was aligned to its corresponding gt16S list using Blast and selected the gt16S sequence of highest alignment. The unweighted average of Blast’s identity score served as the alignment identity.

Explained frequency (Fig. 5B): Overall relative frequencies of groups whose representative 16S rRNA gene sequences aligned to gt16S for a given alignment score.

Erroneous 16S rRNA gene sequences (Fig. 5C): Percentage of 16S rRNA sequences that did not appear in gt16S out of the total number of sequences detected by each method.
