# Supplementary material for: CoSMIC: A hybrid approach for large-scale, high-resolution microbial profiling of novel niches
Source: PLoS One. 2026 Jan 27;21(1):e0340349. doi: 10.1371/journal.pone.0340349 (PMC12844514; doi:10.1371/journal.pone.0340349)
Supplement: Attachment — Submitted filename: CoSMIC_review.pdf [file pone.0340349.s004.pdf]

The paper "CoSMIC – A hybrid approach for large-scale, high-resolution microbial profiling of novel niches" introduces CoSMIC (Comprehensive Small Ribosomal Subunit Mapping and Identification of Communities), a methodological framework designed to enhance microbial community profiling. CoSMIC builds upon the previously developed SMURF approach. According to the authors, CoSMIC offers a practical solution for studying novel ecological niches.

The revisions made by the authors in response to the reviewers' comments are adequate and satisfactory. However, some weaknesses remain that require further clarification and improvement.

1. The full and short titles are the same. Authors should correct this and write the short title.
2. The introduction remains too general and reiterates well-known limitations of standard 16S rRNA profiling (e.g., primer bias, short-read ambiguity) rather than emphasis the specific research problem CoSMIC addresses.
3. The final paragraph in Intruduction section presents partial results instead of clearly stating the research objectives and hypotheses. This should be restructured.
4. Additional citations are needed at the end of sentences in lines 60, 63, and 77 to support key statements.
5. I am not fully convinced by the preparation of the mock mixture. While the rationale is understandable, the analyses would be more reliable if a standardized mock community (e.g., commercially available reference mixtures) had been included.
6. Negative controls were not applied at any stage of the study. This omission makes it impossible to determine to what extent the observed microbial profiles might be influenced by contamination. Based on my own experience, even primer purification methods can significantly affect contamination levels and therefore should be considered.
7. The study does not provide an optimized, ready-to-use primer set. It appears that each user must test multiple primer combinations before identifying the most suitable ones. This lack of standardization reduces the reproducibility and usability of the approach.
8. The authors are reminded that, in accordance with the rules of biological nomenclature (ICZN/ICN), the first time a species is mentioned in the manuscript, the full species name must be provided together with the author citation. In all subsequent mentions of the same species, the genus name should be abbreviated, as required by taxonomic conventions.
9. The use of the Greengenes database is problematic, given that it has not been updated since 2013. Silva is a more appropriate choice.
10. The study does not explain why NCBI was not considered, nor does it compare results obtained with different reference databases. A comparative analysis would strengthen the conclusions and clarify which database is most suitable for CoSMIC.
11. Rarefaction curves for some samples did not reach a plateau, raising concerns about whether sequencing depth was sufficient. This calls into question the reliability of applying CoSMIC to novel and complex samples.
12. Relative abundance estimates are acknowledged as unreliable, but the discussion provides little evidence for how UMIs will solve this issue in practice. This limitation currently reduces the ecological interpretability of CoSMIC results.
13. Claims about harmonizing results across studies and niches are speculative and should be more cautiously stated, given unresolved issues with standardization, database completeness, and contamination controls.
