# Supplementary material for: CoSMIC: A hybrid approach for large-scale, high-resolution microbial profiling of novel niches
Source: PLoS One. 2026 Jan 27;21(1):e0340349. doi: 10.1371/journal.pone.0340349 (PMC12844514; doi:10.1371/journal.pone.0340349)
Supplement: Attachment — Submitted filename: COSMIC point by point rebuttal.pdf [file pone.0340349.s005.pdf]

Response to Reviewer 1

Reviewer's Comment: "Introduction - Discuss alternative methods to the LNA. Could hybridization-based enrichment work? Was it used in the past?"

Answer: We thank the Reviewer for the comment. Indeed, hybridization-based enrichment has been successfully used for the targeted capture of 16S rRNA genes; yet, we suggest that LNA primers offer distinct advantages for our application. We, therefore, added a section on alternative enrichment methods, aiming to justify our choice of LNA primers (for clarity, the section was added to the Discussion).

Added paragraph: Beyond these alternative workflows, researchers may consider other methods for the initial enrichment of full-length 16S rRNA sequences. Hybridization-based enrichment methods, such as SureSelect (Agilent) and xGen Lockdown probes (IDT), have been successfully applied to enrich 16S rRNA genes from complex samples (29, 30). However, we preferred using LNA primers for several reasons: *(a)* their shorter length allows for nearly universal amplification while maintaining specificity through locked nucleic acid modifications (hybridization probes are two to ten times longer than the 10-13 nt of the LNA primers); *(b)* a PCR-based approach is considerably more cost-effective than hybridization capture; and *(c)* the protocol is simpler, faster, and requires only standard PCR equipment, unlike specialized hybridization workflows.

Reviewer's Comment: "172-174 -- unclear sentence"

Answer: We agree that this statement requires clarification. We edited two sentences in the section "Combining results from several variable regions partially overcomes problems in profiling", and hope their combination improves clarity.

First, the term group was defined in the first paragraph of the section:

"The output of SMURF is a set of "groups" and their relative frequencies, where each group contains full-length 16S rRNA genes having an identical sequence over the amplified regions."

For clarity, we have now edited this sentence to specify that members of a "group" differ across the rest of the gene, i.e., the regions that were not amplified:

"The output of SMURF is a set of "groups" and their relative frequencies, where each group contains full-length 16S rRNA genes having an identical sequence over the amplified regions (i.e., these sequences differ across the rest of the 16S rRNA gene, e.g., in regions that were not part of the primer set)."

Second, Figure 2 presents the effect of using six primer pairs while performing SMURF-based profiling using different subsets of primer pairs. Hence, profiling by n+1 primer pairs can exclude sequences that were part of a group when using n regions, based on the reads provided by the added region.

We have edited the sentence highlighted by the Reviewer:

“Therefore, based on the reads from each additional region, full-length 16S rRNA sequences that were once part of a group are being excluded”.

Reviewer's Comment: "180-186 -- Can you provide an explanation/hypothesis for the differences between samples? Why do you think the differences in results are consistent between experiments? If there is no reason to assume consistency, why are Figs 2D-E important?"

Answer: We thank the reviewer for this question. We have understood the Reviewer nomenclature as follows: the “differences between samples” above refer to potential differences between niches (e.g., should soil and plant samples be profiled with different primer pairs), while the question “consisten(cy) between experiments” refers to the differences between measurements within a niche (i.e., should different soil samples collected from the same area be profiled with the same primer pair).

Difference between niches - We argue that primer performance is niche-dependent because different 16S rRNA variable regions are better at resolving the specific taxa that dominate different environments. This statement is well acknowledged.

Consistency among experiments within the same niche: We found that performance is highly consistent *in* a given niche. For example, we examined soil samples and evaluated all measures shown in Figure 2 (ambiguity, mutual species, and Pearson r). For each specific combination of 2, 3, 4, and 5 primer pairs, we calculated the average and variance for each measure across soil samples. Supplementary Figure 18 (see below) displays the variance as a function of the mean, showing a very high correlation. Therefore, the best primer pair chosen based on the mean across soil samples also has the lowest variance, indicating consistent results across soil samples. We performed the same analysis on root and leaf samples, as shown in Supplementary Figure 19.

The following text was added at the end of the Results section, “Combining results from several variable regions partially overcomes problems in profiling”:

Performance across the three measures in Figure 2 is highly consistent within a given niche. For example, the best primer pairs, chosen based on average ambiguity across soil samples, also have the lowest ambiguity variance, indicating consistent results across soil samples (Supplementary Figures 18-19 for soil, root, and leaf).

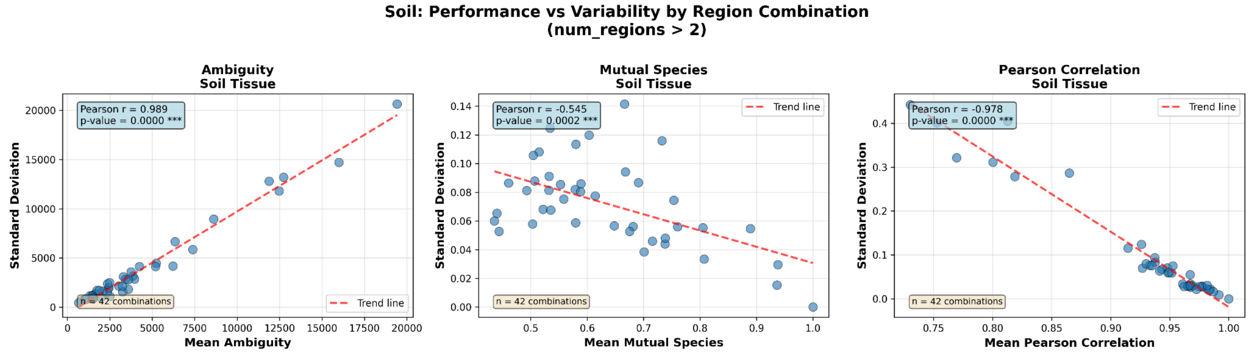

Figure S18: Performance measures of each primer pair combination - the correlation between the mean value across soil samples and their variance. We analyzed all 42 combinations of 2, 3, 4, and 5 primer pairs and calculated Ambiguity (left), Mutual Species (middle), and Pearson correlation (right). For each primer combination, we compared the mean of the relevant measure across 8 soil samples (horizontal axis) to its variance. Red dashed lines indicate linear regression trends. Statistical annotations show Pearson correlation coefficients (r) with corresponding p-values, where significance is indicated as: *** p < 0.001, ** p < 0.01, * p < 0.05, ns = not significant.

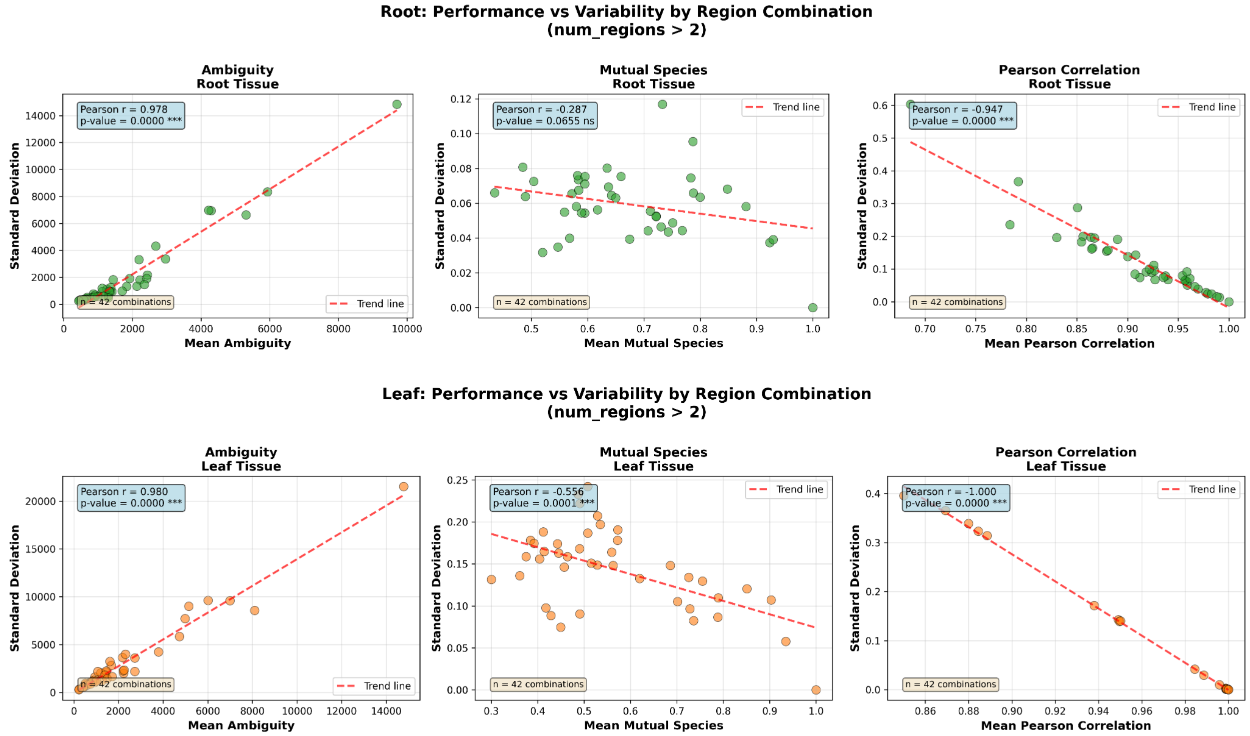

Figure S19: The same as Figure S18, for root (upper panel) and leaf (lower panels)

Reviewer's Comment: "220 -- The measures taken to remove PCR chimeras are not clear enough. How many of the suspected chimeras ended as 'novel sequences'?"

Answer: Several measures were implemented to prevent chimeras from being misclassified as new sequences. These measures are described in the Method section titled “Filtering potential chimeras.” This information was added to the original sentence on line 220. Additionally, *(a)* these steps are documented in the GitHub repository, and *(b)* a Discussion paragraph addresses these chimera detection methods. That paragraph also explains why, even if these methods do not identify every chimeric sequence, there is no impact on downstream microbial profiling results. This is because no short reads support a chimeric sequence, so SMURF will not select such false full-length 16S rRNA sequences. The Discussion section states that only novel sequences identified by SMURF (i.e., via short reads) will be added to external databases, ensuring their validity. For text editing—please see the answer to the LNA pipeline comment below**.**

Reviewer's Comment: "Provide more details in the main text about the LNA system. Add to Figure 1 the LNA primers and their relative place to the other primers. How the transition to LNA affected the efficacy of the PCR?"

Answer: All available details regarding the LNA system appear in the Methods section and are presented below. In addition, we created a new Supplementary figure depicting the locations of LNA primers and refer to it as described below (we preferred to create a new figure because LNAs are first mentioned later in the Results). As for PCR efficiency, regular primers of the same length would have had a much lower Tm, and the reaction would not have occurred. In contrast, LNA amplification was highly effective, resulting in many full-length sequences. These sequences were subsequently detected by short-read sequencing, thereby demonstrating the efficacy of LNA amplification.

LNA primers’ positions figure:

Results section “Enriching the database using long-read sequencing” (former Lines 213-215): The procedure entails a PCR step, utilizing a generic pre-designed multiplexed set of locked nucleic acid (LNA) primers aimed at effectively amplifying the whole 16S rRNA gene (see Methods and Supplementary Figure 17).

Figure S17:

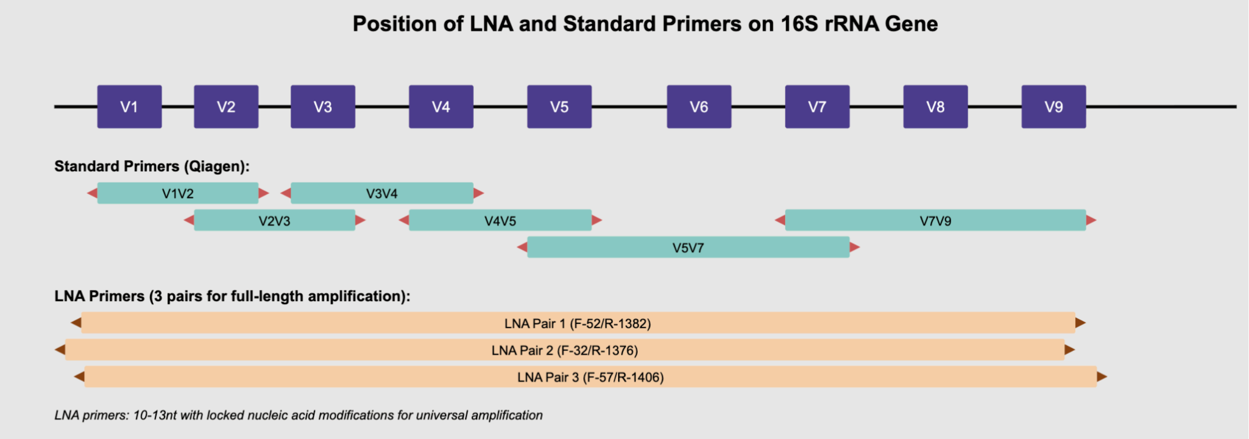

Figure S17. Positions of LNA and standard primers along the bacterial 16S rRNA gene. Schematic representation of variable regions (upper panel) and primer locations for six pairs included in the Qiagen QIAseq panel (middle panel). The lower panel shows the location of our LNA primers, positioned at conserved regions near the gene termini (forward primers at positions 32-57; reverse primers at positions 1376-1406 relative to *E. coli* 16S rRNA).

Mentions of the LNA system in the Methods section:

1) Methods section "Designing LNA primers for full-length 16S rRNA sequences" explains the design process, mentioning the short primer pairs (10-13nt) to maximize amplified genes. It identified three candidate pairs that amplify 1.2M out of 1.4M sequences. We added a remark about the percentage of amplified SILVA sequences in response to another question by the Reviewer.
2) Table 1A lists the specific LNA primer sequences, their positions, and Qiagen catalog numbers. As mentioned in the text, Qiagen considers the identities of the modified nucleotides to be trade secrets; however, our designed primers have Qiagen catalog numbers and can be purchased.
3) Methods subsection "Sample preparation" (under section “Enriching the database with novel 16S rRNA gene sequences”) describes the practical use of LNA primers, including PCR conditions (annealing at 55-58°C) and amplification protocol.

Reviewer's Comment: "LNA primers -- how many bases are LNA? All of them?"

Answer: We agree with the Reviewer that the information about LNA primers is not complete. We provided all available information, i.e., the primers’ sequences and their Qiagen catalog number. The specifics of modified bases are a Qiagen trade secret. Following the Reviewer’s comment, we pushed Qiagen on this issue, yet the best answer we received was that “only a

handful of strategic bases are LNA". We apologize for not being able to answer this question. We added the following sentence to the caption of Table 1:

Table 1. LNA primers. A. Three primer pairs (pairs 1-3) and their location along the *E. coli* 16S rRNA gene. Primers are available via the Qiagen catalog number. The specifics of modified bases are a Qiagen trade secret, yet only a handful of bases are modified. **B.** The number of amplified full-length 16S rRNA genes by each pair and by their intersection. About 360K are amplified by all three pairs, 570K by two pairs, and 250K by just a single pair.

Reviewer's Comment: "Add explanations to the climate classifications in simple words"

Answer: We revised the text as follows (the legend was revised accordingly):

To illustrate this idea and estimate the number of novel sequences that can be detected, we used this approach over pools of samples, each from a different niche: (a) sponges of the species *Spongia officinalis* collected from the Mediterranean coast of France; (b) *S. officinalis* collected from the Mediterranean coast of Israel; (c) plants from the Arava desert in Israel (climate type, BWh defined as hot desert climate with annual rainfall <100mm); and (d) plants from the temperate lowland in Israel (climate type, Csa defined as a Mediterranean climate with hot, dry summers and mild, wet winters).

Reviewer's Comment: "PacBio sequencing is known to have relatively high error rates. What measures were used to exclude sequencing errors as novel sequences? How do the base errors affect the results?"

Answer: We thank the reviewer for raising these important points. We consider the questions of PacBio error rate and its effect on downstream profiling results separately.

<u>Ensuring PacBio sequence quality</u>

We implemented several measures to ensure sequencing errors do not inflate the set of novel sequences:

1. **HiFi/CCS technology**: We used PacBio's circular consensus sequencing (CCS), which achieves >99.5% accuracy by sequencing the same molecule multiple times (this is feasible in our case since the amplicon length is ~1500 bp). We retained only high-fidelity sequences resulting in Q40+ scores.
2. **Quality filtering**: We applied the SMRT analysis software for initial quality filtering, removing low-quality reads and chimeras.
3. **Clustering sequences at 99% similarity**: We clustered sequences at 99% identity using CD-HIT, which collapses sequences differing by 1-2 bases into a single representative, effectively eliminating potential sequencing errors.

The effect of erroneous long-read sequencing results on short-read profiling results

Erroneous full-length 16S rRNA sequences will not appear in SMURF's short-read profiling. We addressed this question in the context of chimeric sequences (see our reply above); the same answer applies to other types of erroneous long-read sequences.

We edited the Results and Methods sections to reflect the measures taken to overcome PacBio errors:

Results section "Enriching the database using long-read sequencing":

These pools were sequenced using PacBio's HiFi circular consensus sequencing (CCS), which generates reads with over 99.5% per-base accuracy by sequencing the same molecule multiple times (Wenger et al., 2019). The resulting reads went through a rigorous quality control process that included chimera removal (see Methods, "Filtering potential chimeras") and strict quality filtering (Q-score >40). To address any remaining sequencing errors and eliminate redundancy, the filtered sequences were then clustered at 99% identity. This clustering step prevents single-base errors or minor variants from artificially increasing the count of new sequences, as they are combined into a single representative. This entire process resulted in approximately 200K high-quality, full-length 16S rRNA sequences (Supplementary Figure 11) with an average length of 1600 bp (Supplementary Figure 12).

We edited the Methods subsection "PacBio sequencing" to reflect quality filtering and error correction:

16S rRNA full-length amplicons obtained from LNA amplification of the microbial communities in Spongia officinalis (France and Israel) and in plants from Israel's lowland and Arava were used as starting templates for the SMRTbell Express Template Prep Kit 2.0 (Product PN. 100-938-900). Sequencing was performed using the PacBio Sequel sequencer. Only circular consensus sequences (CCS) with ≥3 passes and quality scores >Q40 were retained, ensuring >99.5% per-base accuracy. The SMRT(33) analysis software performed additional quality filtering and chimera removal (reads are eliminated if two SMRTbell adaptors are sequenced). To address residual sequencing errors (~0.5%), all sequences were clustered at 99% identity using CD-HIT (see "Calling novel 16S rRNA genes"), which merges sequences differing by minor base errors into single representatives. This combination of high-accuracy CCS reads and 99% clustering prevents sequencing errors from being mistaken for novel sequences.

Reviewer's Comment: "Fig 3b - The difference between the distribution of aligned and unaligned reads from Sponges is suspicious. How do you explain it? Could it be that these are sequencing artifacts?"

Answer: We thank the Reviewer for this comment. We do not believe these differences are related to PacBio sequencing errors (see our response to the previous comment). Furthermore, as stated in the section, 93% of the relative frequency in sponge-associated bacteria detected

by short reads corresponds to novel sequences (compared to 10% in plants). Therefore, we think that the differences in distributions reflect the “novelty” of the sponges’ niche with respect to SILVA, compared to the more “well-known” plant niche.

Reviewer's Comment: "Fig 3c -- add the Sponges to the legend

Answer: Done.

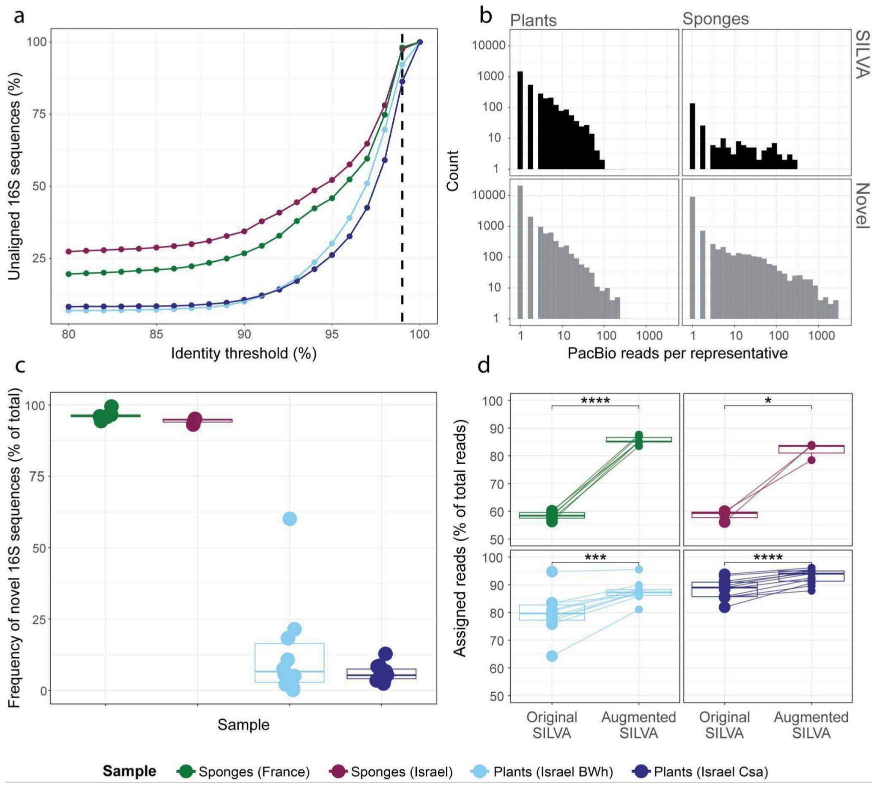

Figure 3. Enriching the database with novel full-length 16S rRNA sequences. We amplified the full-length 16S rRNA gene using pre-designed LNA primers and sequenced the resulting amplicons by PacBio long-read sequencing. Four samples were processed, each containing a pool of samples from a different habitat, i.e., plants from the Arava desert in Israel, characterized by a climate type BWh (defined as hot desert climate with annual rainfall <100mm), according to Koppen climate classification; plants from the lowland region in Israel, characterized by a climate type Csa (defined as a Mediterranean climate with hot, dry summers and mild, wet winters); and pools of samples from the marine sponge *S. officinalis* collected in the Mediterranean Sea along the coast of either France or Israel. **a.** All non-redundant PacBio sequences (Plants BWh - 14852, Plants Csa - 14589, Sponges -France - 6507, Sponges-Israel - 5468) were aligned to SILVA using the CD-HIT algorithm(18). The percentage of sequences that did not appear in SILVA is shown as a function of the identity threshold. The vertical dashed line corresponds to SILVA’s standard threshold for calling novel full-length 16S rRNA sequences (SILVA NR99). Colors indicate different biomes sampled. **b.** Histograms of read counts per 16S rRNA sequence for the plants’ pool (left panels) and the sponges’ pool (right panels). The upper and lower panels show histograms of SILVA and novel sequences, respectively. An identity threshold of 99% was used to call novel sequences with respect to SILVA. **c.** Individual samples from the abovementioned pools were sequenced via short reads and analyzed by SMURF using the augmented database to determine their microbial composition (Sponges-France – 6 samples, Sponges-Israel – 3 samples, Plants (BWh) – 10 samples, Plants (Csa) – 10 samples). The total relative frequency assigned by SMURF to novel 16S rRNA sequences per sample (dots) and across types (columns) is shown. **d.** The fraction of reads aligned to either SILVA or augmented SILVA for each sample depicted in C. Each panel corresponds to a different sample type. To evaluate the statistical significance, a paired t-test was performed for each sample type; * $P<0.05$, *** $P<0.001$, **** $P<0.0001$.

Reviewer's Comment: "268-275 -- The overrepresentation of some sequences may be a result of the amplification process. Are these sequences shorter than the other sequences?"

Answer: We thank the reviewer for this comment. To assess potential PCR bias toward shorter fragments, we examined the association between sequence length and relative abundance. The Pearson correlation between length and read count was negligible ($R = -0.012$, $p = 1.8e^{-5}$).

The following Supplementary figure was added, together with the following sentence in the results section line 259 .

"The correlation between sequence length and the number of reads was negligible, i.e., Pearson $R = -0.012$, $p = 1.8e^{-5}$, thus excluding the possibility of amplification-related length biases (see Supplementary Figure 20)"

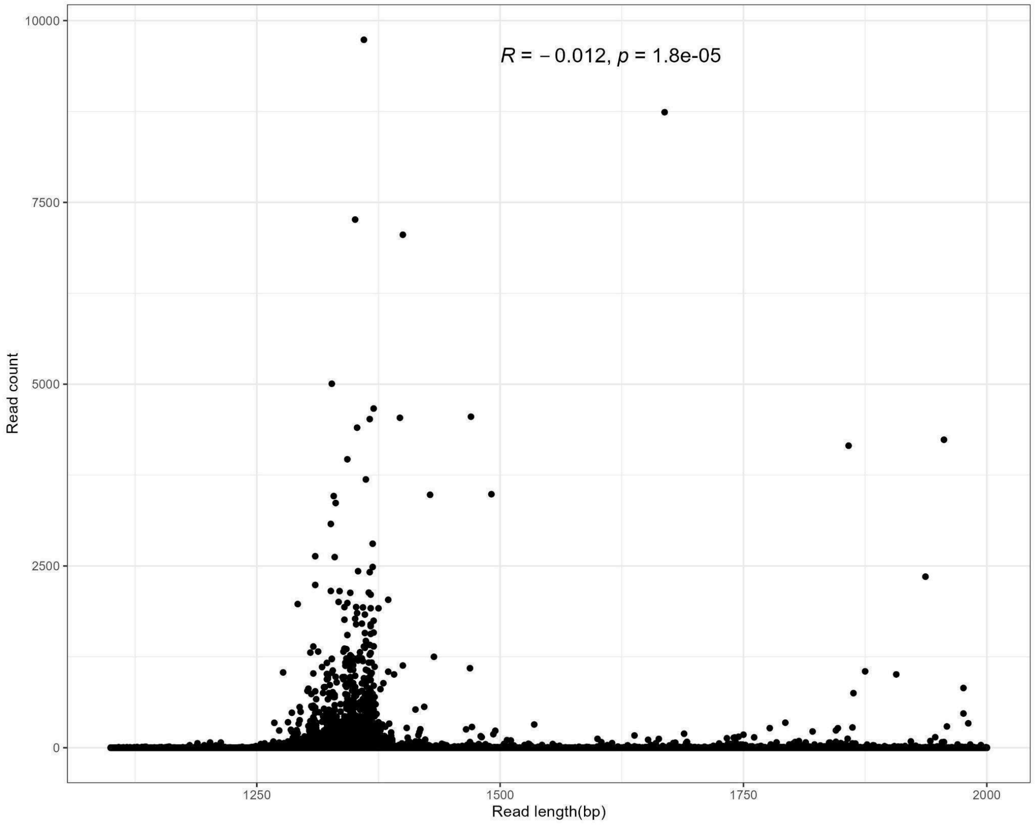

Figure S20. Read count as a function of read length for PacBio sequences retained in the database. Each point corresponds to a 16S rRNA sequence (or a cluster of sequences) retained after PacBio sequencing and subsequent postprocessing and filtering. The figure displays each sequence length vs. its observed count. There is no evidence of an association between sequence length and abundance (Pearson $R = -0.012$, $p = 1.8e^{-5}$).

Reviewer's Comment: "322 - Hymelaea hirsuta -- Do you mean Thymelaea hirsuta? In figure 5 it is written with initial T."

Answer: Corrected.

Reviewer's Comment: "799 -- fill the REF with the reference"

Answer: Corrected:

Revised Text: "The software package (https://github.com/NoamShental/CoSMIC) provides a comprehensive set"

Response to Reviewer 2

Reviewer's Comment: "The full and short titles are the same. Authors should correct this and write the short title."

Answer: Corrected.

Full title: "CoSMIC - A hybrid approach for large-scale, high-resolution microbial profiling of novel niches"

Short title: "CoSMIC: High-resolution profiling of novel niches"

Reviewer's Comment: "The introduction remains too general and reiterates well-known limitations of standard 16S rRNA profiling (e.g., primer bias, short-read ambiguity) rather than emphasizing the specific research problem CoSMIC addresses."

Answer: The introduction was organized following this logic: First, we discuss primer bias and ambiguity caused by short-reads, as noted by the Reviewer. Then, we introduce SMURF as a solution to these issues, and later explain its limitations in non-human-associated niches. This leads to the research problem addressed by CoSMIC. We agree that the structure may be somewhat general; however, we believe it clearly outlines the problem CoSMIC aims to solve.

Reviewer's Comment: "The final paragraph in Introduction section presents partial results instead of clearly stating the research objectives and hypotheses."

Answer: The paragraph before the last in the Introduction follows the logic described in the answer to the previous question: we provide a high-level overview of how CoSMIC addresses the research problem, specifically high-resolution profiling of novel, i.e., underexplored, niches. The results are summarized in a single sentence (as requested by another Reviewer), serving as motivation to adopt a new paradigm in a well-established field. The final paragraph in the Introduction highlights CoSMIC's "selling point," that is, who and where we propose using this approach. We hope this can also be seen as another aspect of our research objectives.

Reviewer's Comment: "Additional citations are needed at the end of sentences in lines 60, 63, and 77 to support key statements."

Answer: Done. The revised text appears below.

(former) Line 60: "Such ambiguity in assigning reads to bacteria often results in lower resolution profiling (Johnson et al., 2019; Schloss, 2021; Edgar, 2018)."

(former) Line 63: [However, current bioinformatic pipelines do not adequately address either problem, regardless of whether they use amplicon sequence variants (ASVs) or operational taxonomic units (OTUs).(Schloss et al., 2021, Johnson et al., 2019)

As for (former) line 77 - the statement claiming that LNAs “provide an almost universal amplification of full-length 16S rRNA sequences” is part of our results.

Reviewer's Comment: "I am not fully convinced by the preparation of the mock mixture. While the rationale is understandable, the analyses would be more reliable if a standardized mock community had been included."

Answer: We thank the reviewer for this comment and acknowledge the importance of benchmarking against a standard community. To address this, we have now analyzed the ZymoBIOMICS Microbial Community DNA Standard (Zymo, Cat. No. D6305) using the full CoSMIC pipeline. The results, shown in Supplementary Figure S21, demonstrate a strong agreement between the observed composition and the theoretical composition provided by the manufacturer (Bray-Curtis dissimilarity = 0.061, Jaccard Index = 1). This confirms that CoSMIC accurately reconstructs the makeup of a well-defined, standardized mock community.

We added the following paragraphs to the Results and Methods sections:

(Results line 371): (To further support our performance, we also successfully validated our pipeline using a commercially available standard mock community, achieving complete accuracy; see Methods and Supplementary Figure 21).

(Methods line 798): To further validate the CoSMIC pipeline, we also analyzed a commercially available mock community, i.e., the ZymoBIOMICS Microbial Community DNA Standard (Zymo Research, Cat. No. D6305). The DNA standard was processed through the same multi-region short-read sequencing pipeline as our custom mock community. The resulting composition was compared with the manufacturer's theoretical specifications (Supplementary Figure 21) and showed high similarity to the prepared community.

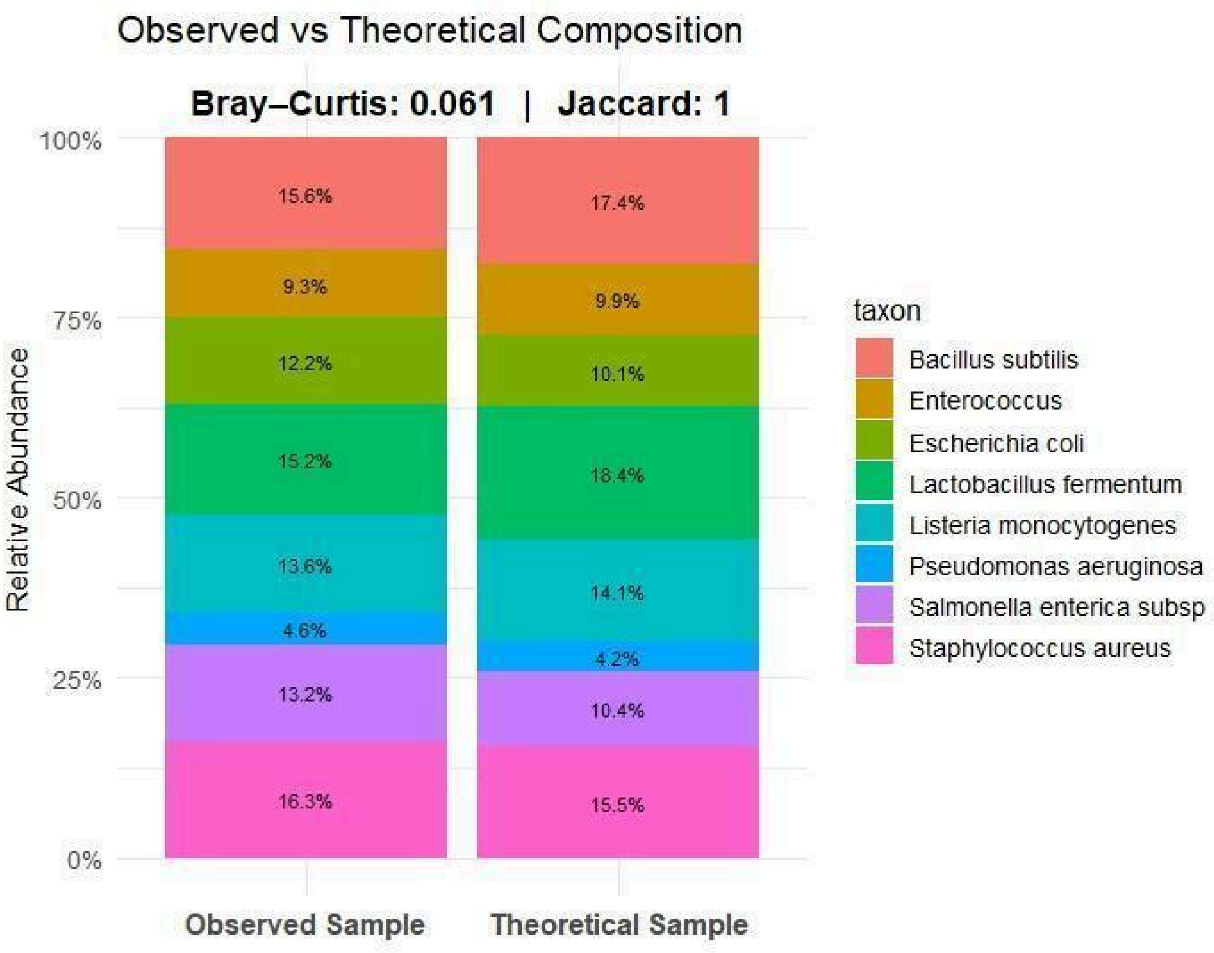

Figure S21. CoSMIC-based profiling of the ZymoBIOMICS microbial community DNA standard. The stacked bar charts compare the microbial composition of the ZymoBIOMICS standard (Zymo Research, Cat. No. D6305) as profiled by the CoSMIC pipeline (Observed Sample) versus the manufacturer's specified composition (Theoretical Sample). Each colored segment indicates the relative abundance of a specific taxon. A Jaccard index of 1.0 indicates that CoSMIC accurately identified all eight expected bacterial species with no false positives. The low Bray-Curtis dissimilarity score of 0.061 reflects a high similarity between the observed and theoretical relative abundances. Together, these findings support the accuracy of the CoSMIC framework against a well-defined, standardized benchmark.

Reviewer's Comment: "Negative controls were not applied at any stage of the study. This omission makes it impossible to determine to what extent the observed microbial profiles might be influenced by contamination."

Answer: We thank the reviewer for raising this essential point on quality control. We apologize for not including this information in the original submission. In fact, no-template controls (NTCs) were processed alongside our samples in all sequencing runs. Our analysis of 11 NTCs

demonstrates the robustness of our workflow against contamination. NTCs yielded a low number of raw reads (median ~23k), and processing them via SMURF resulted in empty profiling results. For example, we performed a detailed analysis of a representative NTC and found that its reads were primarily Illumina adapter dimers. Remaining reads were low-quality short fragments (~35 bp) that matched *Clostridioides difficile*, yet were filtered out prior to SMURF analysis.

We added a Supplementary Results section detailing NTC analysis, and refer to it in the Methods section “Library preparation and sequencing”.

Text added Methods: "No-template controls (NTC) were included in each library preparation and sequencing run. An analysis of these controls is provided in the Supplementary Results, confirming the absence of significant contamination in our profiling results."

Supplementary Results - Analysis of no-template controls (NTCs)

To evaluate the potential effects of reagent and environmental contamination, eleven no-template controls (NTCs) were included and processed alongside the experimental samples across various sequencing libraries. Raw sequencing data from these NTC libraries produced a median of 22,875 reads per sample (Interquartile Range [IQR]: 2,493–43,637; Full Range: 1–169,257). Although raw reads were present, they were effectively removed during our bioinformatics pipeline. A detailed analysis of a representative NTC sample (11,425 reads) showed that the reads consisted of Illumina adapter sequences and very short DNA fragments (~35 bp). Due to their short length and low quality, these sequences were filtered out. As a result, no valid 16S rRNA sequences from any NTC passed to SMURF, leading to empty community profiles for all negative controls. This demonstrates that our workflow is resilient to low-level contamination and that such artifacts do not affect the microbial profiles reported in this study.

Reviewer's Comment: The study does not provide an optimized, ready-to-use primer set. It appears that each user must test multiple primer combinations before identifying the most suitable ones.

Answer: We thank the Reviewer for this comment. While our approach intentionally provides flexibility, we understand the need for more precise guidance. Specifically, we aim to clarify (a) which parts are fixed (long-reads LNAs); (b) those that require selection (primers for short-read sequencing); and (c) the issue of optimization if budget considerations are relevant. As for the latter, we note that there is no intrinsic need for users to test multiple combinations before identifying the most suitable ones.

To clarify these points, we added a Methods section “A practical summary for primer selection in CoSMIC”:

A practical guide to primer selection in CoSMIC

CoSMIC involves two distinct sequencing stages, each with its own primer strategy. This section highlights several practical issues regarding each step.

1. Universal LNA primers for long-read Sequencing (fixed primer set):
The initial database enrichment step uses a pre-designed, fixed set of three universal LNA primer pairs (Table 1). These primers are designed to be niche-independent and should be used without modification to amplify full-length 16S rRNA genes from pooled samples of any new environment.

2. Multi-region primers for short-read sequencing (standard primer set):
For the second stage of profiling individual samples, we recommend using a commercially available, off-the-shelf kit that amplifies multiple non-consecutive regions of the 16S rRNA gene (e.g., the 6-region Qiagen panel or the 9-region Swift panel used in this study). This standardized approach is the most straightforward and robust. The CoSMIC software is designed to work with the output of such kits, but can also be adapted for custom primer sets.

3. Primer subset optimization for short-read sequencing (optional for budget-constrained projects):
For most projects, we recommend using all amplicons provided by a commercial kit, as this maximizes resolution, and the marginal cost of adding primer pairs is often low. However, for very large-scale studies where cost savings are crucial, an optional optimization can be performed. As shown in Figure 2, a user can sequence a small set of representative samples (e.g., 10-20) with the full primer panel. Analyzing the resulting data can then identify a minimal subset of primer pairs (e.g., 3-4 out of the whole set from the kit) that provides sufficient resolution for that specific niche. The large-scale project can then sequence the remaining samples using only this optimized, cost-effective subset of primers.

Reviewer's Comment: "In accordance with the rules of biological nomenclature (ICZN/ICN), the first time a species is mentioned in the manuscript, the full species name must be provided together with the author citation."

Answer: Done.

Reviewer's Comment: "The use of the Greengenes database is problematic, given that it has not been updated since 2013. Silva is a more appropriate choice."

Answer: The Greengenes database was used solely to design our primers for near-universal full-length 16S rRNA gene amplification (SILVA was used as our “baseline” database for short read profiling via SMURF). The design criterion opted for universal amplification of full-length 16S rRNA sequences; therefore, since both Greengenes and SILVA are sufficiently large, they should, in principle, show the same proportion of amplified sequences. Following the Reviewer’s

comment, we calculated the percentage of SILVA sequences amplified by our LNA primers, achieving 84%, compared to 86% in Greengenes, thus confirming robust performance.

We added the sentence below to the Methods section, “Designing LNA primers for full-length 16S rRNA sequences”:

Considering only perfect matches while ignoring thermodynamic considerations(14), we identified three candidate pairs that potentially amplify 1.2M out of the 1.4M sequences, corresponding to 86% of Greengenes sequences (analogously, these primers potentially amplify 84% of SILVA NR99 sequences). Candidate pairs were provided to Qiagen to replace specific nucleotides with locked nucleic acids, resulting in our LNA primer set (Table 1).

Reviewer's Comment: "The study does not explain why NCBI was not considered, nor does it compare results obtained with different reference databases."

Answer: We thank the reviewer for this question regarding our choice of reference database.

In short, our choice of SILVA reflects its status as the community standard for a high-quality, expertly curated 16S rRNA gene database. While the NCBI nucleotide database is significantly larger, it is a general-purpose repository. As a result, it contains a high level of redundancy, many partial sequences, and inconsistent annotations, making it computationally challenging and less suitable for the high-resolution taxonomic profiling CoSMIC aims to achieve. SILVA, on the other hand, is specifically optimized for this purpose, offering a robust and well-structured taxonomic system framework.

More importantly, a key strength of the CoSMIC methodology is that it directly tackles the limitations of any “initial” database. By generating and incorporating novel, niche-specific full-length sequences, CoSMIC builds an "augmented" database that is more comprehensive for the specific environment being studied. This enrichment step makes the final analysis much less reliant on the completeness of the initial baseline database compared to standard methods. While we selected SILVA as the best-practice starting point, the CoSMIC framework is designed to improve any database, and our software can be adapted by users to include other databases if they choose.

Reviewer's Comment: "Rarefaction curves for some samples did not reach a plateau, raising concerns about whether sequencing depth was sufficient."

Answer: We agree with the Reviewer that deeper long-read sequencing would have detected more new sequences. However, we emphasize that 91-97% of sequences are associated with fewer than 10 reads, i.e., these are presumably rare sequences. We believe that current long-read sequencing was sufficient to capture the major sequences since (a) Novel sequences detected by PacBio were attributed to >93% of relative abundance in samples as detected by

short-read sequencing (Fig. 3C); (b) Read assignment improved by 24% (sponges) and 4-8% (plants) (Fig. 3D); and (c) All abundant sequences (>100 reads) found by PacBio were captured by short-reads.

We edited the Result section to clarify these points (line 300):

Another observation is that 91% and 97% of the novel sequences for sponges and plants, respectively, appear in less than 10 PacBio reads (Fig. 3B). Although rarefaction curves plotted over these pools do not reach a plateau for either sponges or plants (Supplementary Figure 13), indicating extensive rare biosphere diversity, the captured sequences account for >90% of community abundance in individual samples. Deeper sequencing would have identified additional rare taxa (<10 reads), which probably would have closed the remaining gap in unaligned reads.

Reviewer's Comment: "Relative abundance estimates are acknowledged as unreliable, but the discussion provides little evidence for how UMIs will solve this issue in practice."

Answer: The idea is that tagging individual template molecules before amplification would allow for computational correction of differential amplification efficiencies. The Discussion section mentions this UMI idea as work in progress. We do not claim to have solved it. We edited the following Discussion sentence:

To overcome this problem and allow for accurate estimates of relative frequencies, we aim to utilize unique molecular identifiers (UMIs) as part of the CoSMIC sample preparation, similar to our former quantification method (31). UMI tagging individual template molecules prior to amplification may allow downstream computational correction of varying amplification efficiencies.

Reviewer's Comment: "Claims about harmonizing results across studies and niches are speculative and should be more cautiously stated, given unresolved issues with standardization, database completeness, and contamination controls."

Answer: We agree that there are many other caveats that CoSMIC does not aim to address, which hinder harmonization. We therefore tempered the statement.

In conclusion, using CoSMIC will solve issues caused by standard profiling methods, help infer species functions and biological significance, and hopefully pave the way for harmonizing results across studies from the same or different niches. We believe this framework, which enables low-cost, high-resolution microbial profiling of any niche, offers a modular and optimized solution for the evolving landscape of microbiome research.

Response to Editor's Journal Requirements

Comment 1: PLOS ONE style requirements

Editor's Comment: "Please ensure that your manuscript meets PLOS ONE's style requirements, including those for file naming."

Answer: Done

Comment 2: Field permits information

Editor's Comment: "In your Methods section, please provide additional information regarding the permits you obtained for the work. Please ensure you have included the full name of the authority that approved the field site access and, if no permits were required, a brief statement explaining why."

Answer: We have added a Methods subsection titled “Field collection permits”. This section includes the full names of the issuing authorities and the corresponding permit numbers for samples collected in both Israel and France. We have also included a statement clarifying that no permits were required for soil sampling, in accordance with local regulations.

Field Collection Permits

All samples were collected in accordance with local and national regulations.

Israel: Permits for collecting plant samples were granted by the Israel Nature and Parks Authority (permit numbers: 2021/42189, 2021/42190). Sponge samples from Israel's coast (off Herzliya, 32.17710°N, 34.63306°E) were collected under permit number 2021/42929, also issued by the Israel Nature and Parks Authority. Voucher specimens of the collected Israeli sponge material have been deposited in the Steinhardt Museum of Natural History at Tel Aviv University. No specific permits were required for collecting soil samples, which complies with Israeli regulations for microbial sampling from public lands.

France: Sponge samples were collected within the Calanques National Park (near Marseille, 43°12'38.0"N 5°20'12.3"E). These collections were authorized under permit Arrêté N° 47 DU 29 JANVIER 2020, granted to the Institut Pythéas for scientific sampling by the Préfet de la Région Provence-Alpes-Côte d'Azur.

Comment 3: Funding disclosure statement

Editor's Comment: "Please state what role the funders took in the study. If the funders had no role, please state: 'The funders had no role in study design, data collection and analysis, decision to publish, or preparation of the manuscript.'"

Answer: The sentence “The funders had no role in study design, data collection and analysis, decision to publish, or preparation of the manuscript” was added to the acknowledgments section.

Comment 4: LaTeX template requirement

Editor's Comment: "Please update your submission to use the PLOS LaTeX template."

Answer: Done

Comment 5: Review recommended citations

Editor's Comment: "If the reviewer comments include a recommendation to cite specific previously published works, please review and evaluate these publications to determine whether they are relevant and should be cited."

Answer: Done. We have evaluated all reviewer-recommended citations and have included them.

Comment 6: Reference list verification

Editor's Comment: "Please review your reference list to ensure that it is complete and correct. If you have cited papers that have been retracted, please include the rationale for doing so in the manuscript text, or remove these references."

Answer: Done.
