# Supplementary material for: CoSMIC: A hybrid approach for large-scale, high-resolution microbial profiling of novel niches
Source: PLoS One. 2026 Jan 27;21(1):e0340349. doi: 10.1371/journal.pone.0340349 (PMC12844514; doi:10.1371/journal.pone.0340349)
Supplement: Attachment — Submitted filename: PONE-D-25-22924_reviewer_Comments.docx [file pone.0340349.s003.docx]

Introduction - Discuss alternative methods to the LNA. Could hybridization-based enrichment work? Was it used in the past?

172-174 – unclear sentence

180-186 – Can you provide an explanation/hypothesis for the differences between samples? Why do you think the differences in results are consistent between experiments? If there is no reason to assume consistency, why are Figs 2D-E important?

220 – The measures taken to remove PCR chimeras are not clear enough. How many of the suspected chimeras ended as "novel sequences"?

Provide more details in the main text about the LNA system. Add to Figure 1 the LNA primers and their relative place to the other primers. How the transition to LNA affected the efficacy of the PCR?

LNA primers – how many bases are LNA? All of them?

Add explanations to the climate classifications in simple words

PacBio sequencing is known to have relatively high error rates. What measures were used to exclude sequencing errors as novel sequences? How do the base errors affect the results?

Fig 3b - The difference between the distribution of aligned and unaligned reads from Sponges is suspicious. How do you explain it? Could it be that these are sequencing artifacts? How do role out that these are indeed changes in the abundance of the sequence and not artifacts? Good control will be the use of the short-read sequencing (a different method) to evaluate the results. How many novel sequences were confirmed by the short-read sequencing?

Fig 3c – add the Sponges to the legend

268-275 – The overrepresentation of some sequences may be a result of the amplification process. Are these sequences shorter than the other sequences? Short sequences tend to be better amplified.

322 - *Hymelaea hirsuta* – Do you mean Thymelaea hirsuta? In figure 5 it is written with initial T.

799 – fill the REF with the reference
